# Suppression of melanoma by mice lacking MHC-II: Mechanisms and implications for cancer immunotherapy

**DOI:** 10.1084/jem.20240797

**Published:** 2024-10-29

**Authors:** Hexin Shi, Dawson Medler, Jianhui Wang, Rachel Browning, Aijie Liu, Sara Schneider, Claudia Duran Bojorquez, Ashwani Kumar, Xiaohong Li, Jiexia Quan, Sara Ludwig, James J. Moresco, Chao Xing, Eva Marie Y. Moresco, Bruce Beutler

**Affiliations:** 1 Center for the Genetics of Host Defense, University of Texas Southwestern Medical Center, Dallas, TX, USA; 2 Eugene McDermott Center for Human Growth and Development, University of Texas Southwestern Medical Center, Dallas, TX, USA

## Abstract

Immune checkpoint inhibitors interfere with T cell exhaustion but often fail to cure or control cancer long-term in patients. Using a genetic screen in C57BL/6J mice, we discovered a mutation in host *H2-Aa* that caused strong immune-mediated resistance to mouse melanomas. *H2-Aa* encodes an MHC class II α chain, and its absence in C57BL/6J mice eliminates all MHC-II expression. *H2-Aa* deficiency, specifically in dendritic cells (DC), led to a quantitative increase in type 2 conventional DC (cDC2) and a decrease in cDC1. *H2-Aa*–deficient cDC2, but not cDC1, were essential for melanoma suppression and effectively cross-primed and recruited CD8 T cells into tumors. Lack of T regulatory cells, also observed in *H2-Aa* deficiency, contributed to melanoma suppression. Acute disruption of H2-Aa was therapeutic in melanoma-bearing mice, particularly when combined with checkpoint inhibition, which had no therapeutic effect by itself. Our findings suggest that inhibiting MHC-II may be an effective immunotherapeutic approach to enhance immune responses to cancer.

## Teaser

Shi et al. use forward genetics and identify a null allele of *H2-Aa* that enhances cDC2 cross-priming of CD8 T cells, enabling mice to suppress malignant melanoma growth. This work suggests host MHC-II as a potential target for cancer immunotherapy.

## Introduction

Through studies of somatic mutations and chromosome rearrangements that give rise to cancer cells, the majority of driver oncogenes and oncogenic pathways have been identified. Germline mutations also create either dominant or recessive oncogenic alleles, expressed in all somatic cells, that cause cancer susceptibility in families. In contrast, hypomorphic variant alleles conferring protection against cancer are practically unknown. Identifying mechanisms of resistance conferred by such variants may reveal important molecular targets for cancer therapy. Discovery of de novo cancer resistance mutations in humans is practically impossible, but germline mutagenesis in mice may create cancer resistance alleles that can be identified with high confidence in real time via automated meiotic mapping (AMM) (Wang et al., 2015; Xu et al., 2021). We initiated a forward genetic screen for host-mediated cancer resistance in third-generation (G3) C57BL/6J mice with germline mutations induced by N-ethyl-N-nitrosourea (ENU). To date, a total of 23,210 mice from 551 pedigrees, bearing 31,445 non-synonymous coding/splicing changes within 13,596 genes, have been screened for reduced growth or elimination of engrafted B16F10 melanoma tumors. We hypothesized that the screen might identify host genes supporting tumor metabolic requirements, vascularization, or invasive potential, as well as mutations augmenting host immune responses to cancer cells. Here, we describe a deleterious mutation of *H2-Aa*, the single gene encoding the major histocompatibility complex (MHC) class II (MHC-II) α chain in C57BL/6J mice, which enabled mice to suppress melanoma growth.

MHC-II complexes each consist of an α chain and a β chain and are primarily expressed on the surface of antigen-presenting cells (APCs) such as dendritic cells (DC), macrophages, and B cells, where their function is to present exogenously derived peptides (proteins or fragments of proteins from outside the cell) to CD4^+^ T cells and initiate an immune response against specific pathogens (Neefjes et al., 2011). In addition, MHC-II complexes on DC and thymic epithelial cells operate in the absence of microbial infection to present self-antigens that permit CD4^+^ T cell ontogeny (positive selection) and promote CD4^+^ T cell tolerance (negative selection). DC MHC-II complexes also perform this function in the periphery to maintain both CD4 and CD8 T cell tolerance to self-antigens (Leventhal et al., 2016; Muth et al., 2012; Wohn et al., 2020; Zhou et al., 2023). CD4^+^ T cells are greatly reduced or absent in mice and humans deficient in MHC-II (Cosgrove et al., 1991).

Conventional dendritic cells (cDC) are categorized into two primary subsets: type 1 cDC (cDC1) and type 2 cDC (cDC2). cDC1, marked by the presence of XCR1, TLR3, CADM1, and CLEC9a, specialize in cross-presenting antigens to CD8 T cells (displaying peptide epitopes derived from extracellular antigens on MHC-I (Colbert et al., 2020) and promoting Th1 cell development. In contrast, cDC2, characterized by the expression of SIRPα (CD172a) in humans and CD11b in mice, are effective in activating CD4 T cells, especially Th2 or Th17 cells (Cabeza-Cabrerizo et al., 2021; Wculek et al., 2020). Previous work suggested that cDC1 are exclusively responsible for cross-priming antitumor CD8 T cells (activation of CD8 T cells by cross-presented epitopes) (Cancel et al., 2019; Ferris et al., 2020; Gardner et al., 2020). However, by investigating the ability of mice with an *H2-Aa* mutation to suppress melanoma growth, we demonstrated that in mice lacking MHC-II, cDC2 are increased in number; display enhanced cross-presentation, costimulatory molecule expression, and cross-priming; and have an activated APC gene signature. These characteristics were accompanied by a reduction of T regulatory cells (Treg) and an increase of activated CD8 T cells within tumors, which resulted in robust suppression of melanoma growth that was not apparent when only cDC1 lacked *H2-Aa*.

## Results

### *H2-Aa* mutation in mice confers the ability to inhibit the growth of engrafted melanoma

**Figure 1.**
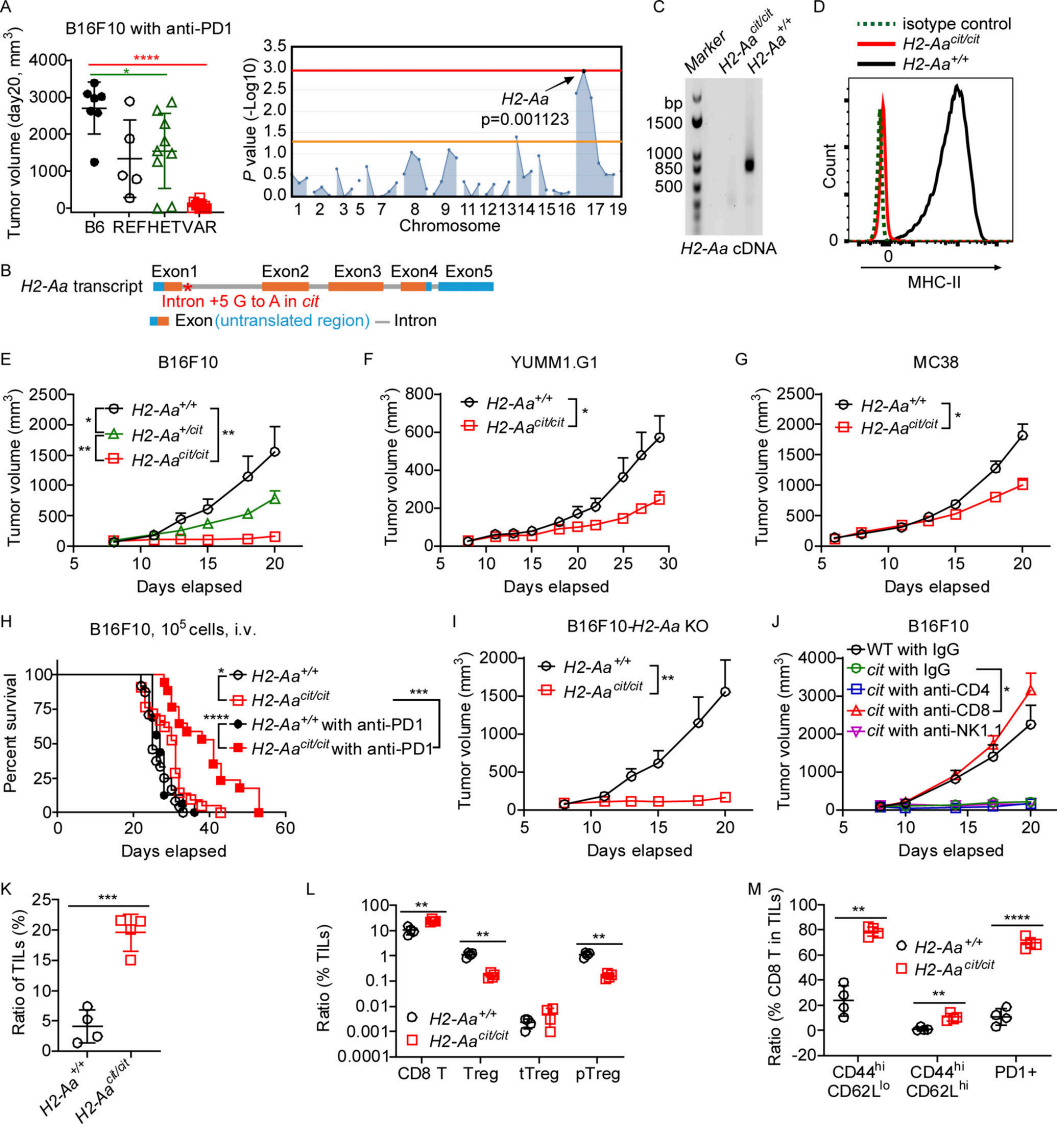
**Mice with the *citation (cit)* allele of *H2-Aa* strongly inhibited melanoma growth. (A)** Left panel: B16F10 melanoma volume on day 20 post-injection s.c. of B16F10 cells into the flank of third generation (G3) descendants of a single G1 male mouse, with REF (+/+), HET (+/mutant), or VAR (mutant/mutant) genotypes for *H2-Aa* (*n* = 7 B6, 5 REF, 9 HET, 10 VAR). Right panel: Manhattan plot showing −log_10_(P values) (Y axis) plotted versus chromosomal positions of mutations (X axis) identified in the G1 founder of the affected pedigree using a recessive model of inheritance. Horizontal red or orange lines represent thresholds of P = 0.05 with or without Bonferroni correction, respectively. The strongest mutation–phenotype association is for a mutation in *H2-Aa*. **(B)** *H2-Aa* transcript diagram showing the location of the *citation* mutation (red asterisk), a G to A transition of the fifth nucleotide of intron 1. Corresponds to 1,128-bp NCBI reference sequence NM_010378.3. **(C)** RT-PCR analysis of *H2-Aa* using primers complementary to sequences in exons 1 and 4. No *H2-Aa* cDNA could be detected in *H2-Aa*^*cit/cit*^ splenocytes. **(D)** WT or *H2-Aa*^*cit/cit*^ splenic B cell (CD19^+^) MHC-II surface expression detected by flow cytometry. **(E–G)** Tumor growth curves of B16F10 melanoma (E) (*n* = 7 +/+, 22 *+/cit*, 9 *cit/cit*), YUMM1.G1 melanoma (F) (*n* = 11 +/+, 14 *cit/cit*), and MC38 colon carcinoma (G) (*n* = 10 +/+, 8 *cit/cit*) after s.c. inoculation on day 0 into the flank of mice. No PD1 antibody was administered. **(H)** Survival curves of mice after i.v. inoculation with B16F10 melanoma on day 0. Mice were intraperitoneally (i.p.) injected with anti-PD1 or vehicle (PBS) twice per week till the end of the experiment (death or euthanasia) (*n* = 16–24 per group). **(I)** Tumor growth curve of B16F10 melanoma in which *H2-Aa* was knocked out (KO) after s.c. inoculation on day 0 into the flank of mice (*n* = 7 +/+, 9 *cit/cit*). **(J)** Tumor growth curve in the presence of cell depleting antibodies. B16F10 cells were injected s.c. on day 0 into the flank of mice. Anti-CD4, anti-CD8, anti-NK1.1, or control IgG, were injected i.p. into *H2-Aa*^*cit/cit*^ (*cit*) mice on days 0, 3, 6, 9, 12, and 15 after tumor inoculation to deplete the corresponding cells (*n* = 4 per group). **(K–M)** Frequency of tumor infiltrating lymphocytes (K), CD8 T cells, Treg, tTreg, and pTreg (L), and the phenotype of CD8 T cells (M) in B16F10 tumors collected on day 13 after B16F10 inoculation (*n* = 4 per group). Data points represent individual mice (A and K–M). Data are representative of one experiment (A) or two independent experiments (C–M). WT littermates (C–M) and WT C57BL/6J mice from JAX (A) were used as controls. Error bars indicate SD (A left panel, K–M) or SEM (E–G, I, and J). P values were determined by Student’s *t* test (A left panel, K–M), two-way ANOVA with post-hoc Tukey test (E–G, I, and J), or log-rank test (H). *P < 0.05; **P < 0.01; ***P < 0.001; ****P < 0.0001.

G3 mice with ENU-induced germline mutations were screened for cancer suppression. In this screen, each G3 mouse was injected subcutaneously with 2 × 10^5^ B16F10 melanoma cells, and a checkpoint inhibitor (anti-PD1 antibody) was administered on days 5, 8, and 11 after tumor cell inoculation. Tumor volume was measured on days 13 and 20. Mutations associated with reduced or absent tumor growth were identified using AMM (Wang et al., 2015; Xu et al., 2021). A putative null allele in *H2-Aa* enabled mice treated with anti-PD1 to suppress the growth of engrafted B16F10 melanoma cells. Several homozygotes entirely resolved their tumors and heterozygotes also significantly slowed melanoma growth as compared with wild-type (WT) controls (Fig. 1 A). We named the phenotype *citation* (*cit*). *H2-Aa* encodes an MHC class II α chain orthologous to human HLA-DQA1. The *cit* allele was predicted to impair the splicing of exon 2, resulting in aberrant *H2-Aa* mRNA maturation (Fig. 1 B). Indeed, the *cit* allele was effectively null, fully prevented *H2-Aa* mRNA splicing, and no mature *H2-Aa* mRNA nor MHC-II protein complex could be detected in *H2-Aa*^*cit/cit*^ splenocytes (Fig. 1, C and D).

**Figure S1.**
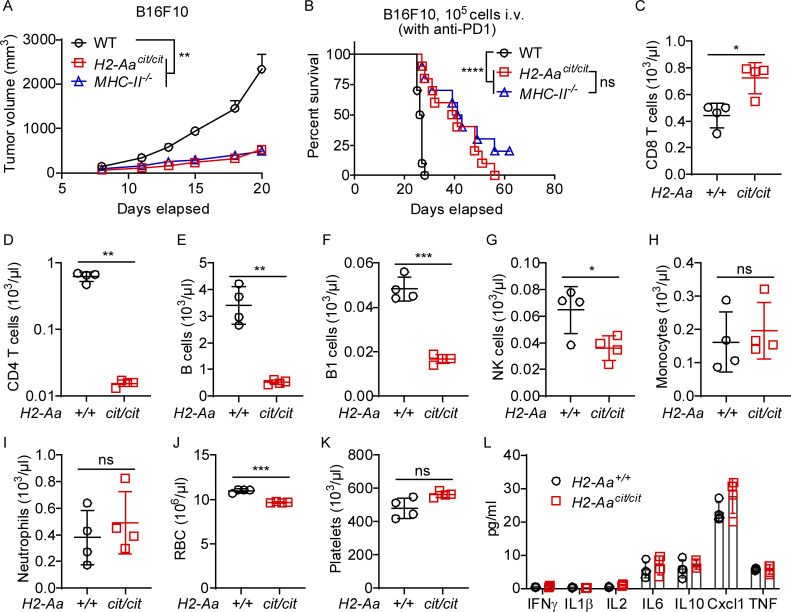
***MHC-II* KO recapitulated *H2-Aa***^***cit/cit***^ **tumor inhibition and phenotyping of *H2-Aa***^***cit/cit***^ **mice. (A)** Tumor growth curves of B16F10 melanoma after s.c. inoculation on day 0 into the flank of mice (*n* = 7 or 8 per group). **(B)** Survival curves of mice after i.v. inoculation with B16F10 melanoma on day 0. Mice were intraperitoneally (i.p.) injected with anti-PD1 twice per week till the end of the experiment (death or euthanasia) (*n* = 10 per group). **(C–K)** CD8 T cells (C), CD4 T cells (D), B cells (E), B1 cells (F), NK cells (G), monocytes (H), neutrophils (I), red blood cells (RBC, J), and platelets (K) in *H2-Aa*^*cit/cit*^ mice and WT littermates. Data were collected by flow cytometric analysis with counting beads (C–I). Data were obtained using a Hemavet 950 (J and K). **(L)** ELISA analysis of cytokines in the peripheral blood plasma of WT and *H2-Aa*^*cit/cit*^ mice. Data are representative of two independent experiments (A–K). WT C57BL/6J mice from JAX (A and B) and WT littermates (C–L) were used as controls. Error bars indicate SEM (A) or SD (C–L). P values were determined by two-way ANOVA with post-hoc Tukey test (A), log-rank test (B), or Student’s *t* test (C–L). Each symbol represents an individual mouse (C–L). *n* = 4 per group (C–L). *P < 0.05; **P < 0.01; ***P < 0.001; ****P < 0.0001; ns, not significant.

Even without anti-PD1, B16F10 tumor growth was significantly inhibited in *cit* homozygotes and heterozygotes, suggesting a semidominant (additive) effect of the mutation (Fig. 1 E). *cit* homozygotes also significantly inhibited the growth of YUMM1.G1 tumors (Meeth et al., 2016), which are C57BL/6J melanoma cells with defined genetic alterations (Fig. 1 F). Similarly, MC38 mouse colon carcinoma tumors were significantly inhibited in *cit* homozygotes as well (Fig. 1 G), indicating that *cit* mice exhibited a broad-spectrum tumor inhibitory effect. We wished to determine whether cooperativity between anti-PD1 and *cit* could be detected. Because *cit* caused elite resistance to B16F10 administered subcutaneously, the effect of anti-PD1 was measured in *H2-Aa*^*+/+*^ and *H2-Aa*^*cit/cit*^ mice that had received intravenously injected B16F10 (Fig. 1 H). Under these conditions, cooperativity was readily detectible, but only in homozygotes. *MHC-II* knockout (KO) mice reproduced the *cit* phenotype (Fig. S1, A and B). To be sure that resistance to melanoma was not caused by a failure to negatively select T cells specific for peptides derived from H2-Aa, leading to CD8 T cell recognition of non-mutated H2-Aa peptides presented by tumor cells, we knocked out *H2-Aa* in B16F10 melanoma cells and showed that *H2-Aa*^*cit/cit*^ mice rejected the KO cells as well as they rejected WT B16F10 (Fig. 1 I).

Both CD8 T cells and NK cells are critical for controlling tumor growth (Mellman et al., 2011), so we investigated their contributions to the antitumor response in *H2-Aa*^*cit/cit*^ mice. Tumor resistance in *H2-Aa*^*cit/cit*^ mice was entirely dependent upon CD8 T cells, which were numerically increased in *H2-Aa*^*cit/cit*^ mice (Fig. S1 C), in that antibody-mediated CD8 T cell depletion in vivo permitted unrestricted tumor growth (Fig. 1 J). On the other hand, depletion of NK or CD4^+^ T cells had no such effect (Fig. 1 J). Tumor-infiltrating leukocytes (TIL) are heterogeneous CD45^+^ cell populations including both myeloid and lymphoid cells, which play a role in innate and adaptive immunity (Zhang and Zhang, 2020). More TILs (CD45^+^) were observed in tumor samples from *H2-Aa*^*cit/cit*^ than from WT mice (Fig. 1 K). Among them, more CD8 T cells and fewer Treg existed in *H2-Aa*^*cit/cit*^ than in WT mice (Fig. 1 L). Tregs are divided into thymus-derived Treg (tTreg) and peripherally derived Treg (pTreg) according to their differentiation sites (Shevach and Thornton, 2014; Yadav et al., 2012). We noticed few tTreg infiltrated the tumor microenvironment, whereas a large number of pTreg accumulated in melanoma growing in WT mice, but this accumulation was reduced in melanoma growing in *H2-Aa*^*cit/cit*^ mice (Fig. 1 L). Additionally, a higher proportion of activated CD8 T cells (CD44^hi^ CD62L^lo^) and memory CD8 T cells (CD44^hi^ CD62L^hi^) were observed within tumors in *H2-Aa*^*cit/cit*^ mice compared with WT mice (Fig. 1 M). Furthermore, these CD8 T cells exhibited increased PD1 expression in *H2-Aa*^*cit/cit*^ mice (Fig. 1 M), aligning with the observed synergy with anti-PD1 treatment (Fig. 1 H). We measured proinflammatory cytokines in the peripheral blood of *H2-Aa*^*cit/cit*^ mice and found them comparable with those in WT controls (Fig. S1 L). Altogether, the higher frequency of activated CD8 T cells and lower frequency of pTreg among TILs suggested that immune effector function was enhanced in *H2-Aa*^*cit/cit*^ mice.

### cDC2 lacking *H2-Aa* are necessary for tumor suppression in *H2-Aa*^*cit/cit*^ mice

**Figure 2.**
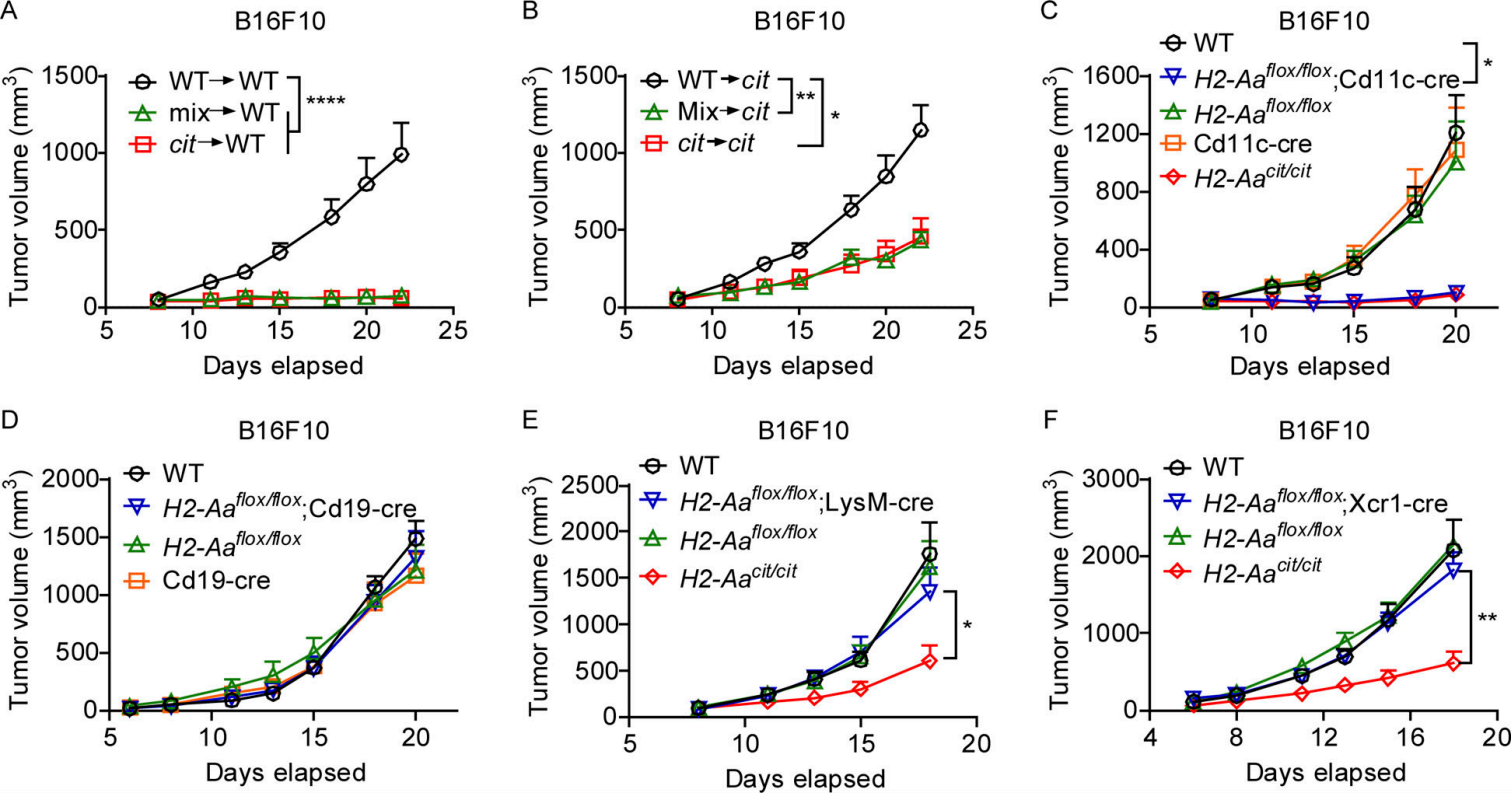
***H2-Aa*–deficient cDC2 are necessary for melanoma inhibition in *H2-Aa***^***cit/cit***^ **mice.** Tumor growth curves of B16F10 melanoma after s.c. inoculation on day 0 into the flank of mice. No PD1 antibody was administered. **(A and B)** Lethally irradiated WT or *H2-Aa*^*cit/cit*^ *(cit)* recipients of WT (CD45.1), or *H2-Aa*^*cit/cit*^ (*cit*, CD45.2), or a 1:1 mixture of *H2-Aa*^*cit/cit*^ and WT bone marrow were inoculated with B16F10 cells 12 wk after bone marrow transfer (*n* = 7 or 8 recipients per group). **(C–F)** Mice in which *H2-Aa* was deleted in (C) DC (*H2-Aa*^*flox/flox*^;Cd11c-cre), (D) B cells (*H2-Aa*^*flox/flox*^;Cd19-cre), (E) macrophages (*H2-Aa*^*flox/flox*^;LysM-cre), or (F) cDC1 (*H2-Aa*^*flox/flox*^;Xcr1-cre). (C–F, *n* = 5–10 mice per group). *H2-Aa*^*cit/cit*^;Xcr1-cre mice in F were checked by flow cytometric analysis to confirm the absence of undesired *H2-Aa* KO in B cells (pre-experiment) and cDC2 cells (post-experiment) to exclude any *H2-Aa* germline deletion due to Xcr1-cre leakage (Ferris et al., 2020; Lança et al., 2022; Wohn et al., 2020). Data are representative of two independent experiments (A–F). WT C57BL/6J mice from JAX (C–F) were used as controls. Error bars indicate SEM (A–F). P values were determined by two-way ANOVA with post-hoc Tukey test (A–F). *P < 0.05; **P < 0.01; ****P < 0.0001.

MHC-II mutations impair CD4 T cell and B cell survival (Brocker, 1997; Labrecque et al., 1999), also evident in *cit* (*H2-Aa*^*cit/cit*^) mice (Fig. S1, D and E). To determine whether the tumor suppression phenotype witnessed in *cit* mice is intrinsic or extrinsic to the hematopoietic compartment, we examined B16F10 growth in bone marrow chimeric mice. WT recipients (CD45.1) of *H2-Aa*^*cit/cit*^ (CD45.2) bone marrow were resistant to B16F10 melanoma growth, even when donor cells consisted of a 1:1 mixture of WT (CD45.1) and *H2-Aa*^*cit/cit*^ (CD45.2) bone marrow cells (BMC) (Fig. 2 A). These results suggested that alteration(s) of hematopoietic stem cells (HSC) or their differentiated progeny from *H2-Aa*^*cit/cit*^ mice conferred resistance to melanoma in the recipients. The reciprocal bone marrow transplantation experiment, in which *H2-Aa*^*cit/cit*^ recipients (CD45.2) of WT (CD45.1) bone marrow failed to inhibit B16F10 melanoma growth, confirmed that the presence of the *cit* mutation in the hematopoietic compartment confers the ability to suppress melanoma growth (Fig. 2 B).

We constructed a conditional KO allele of *H2-Aa* (*H2-Aa*^*flox*^), in which loxP sites were inserted flanking exon 1 of *H2-Aa*, to delete *H2-Aa* in different cell populations derived from HSC. CD11c-cre–mediated *H2-Aa* KO in DC mimicked the *cit* phenotype, providing strong support for a DC-intrinsic mechanism of melanoma rejection (Fig. 2 C). DC-specific Cre expression (Caton et al., 2007) expectedly abolished MHC-II expression not only in DC but also in the majority of B cells (Merkenschlager et al., 2019). However, neither B cell- (CD19-cre) (Rickert et al., 1997) nor macrophage-specific (LysM-cre) (Clausen et al., 1999) *H2-Aa* KO resulted in inhibition of B16F10 melanoma growth (Fig. 2, D and E). To assess whether *H2-Aa*–deficient cDC1 mediated the tumor suppressive effect observed in *H2-Aa*^*cit/cit*^ mice, we utilized an Xcr1-cre driver that is largely restricted to the cDC1 lineage (Ferris et al., 2020; Lanca et al., 2022; Wohn et al., 2020). Surprisingly, with respect to the accepted role of cDC1 in CD8 T cell cross-priming, mice with *H2-Aa*–deficient cDC1 could not inhibit B16F10 melanoma growth at all (Fig. 2 F). Together, these results strongly suggest that *H2-Aa*–deficient cDC2 are critical for melanoma inhibition in *H2-Aa*^*cit/cit*^ mice.

### *H2-Aa*^*cit/cit*^ cDC2 have increased cross-priming activity

**Figure 3.**
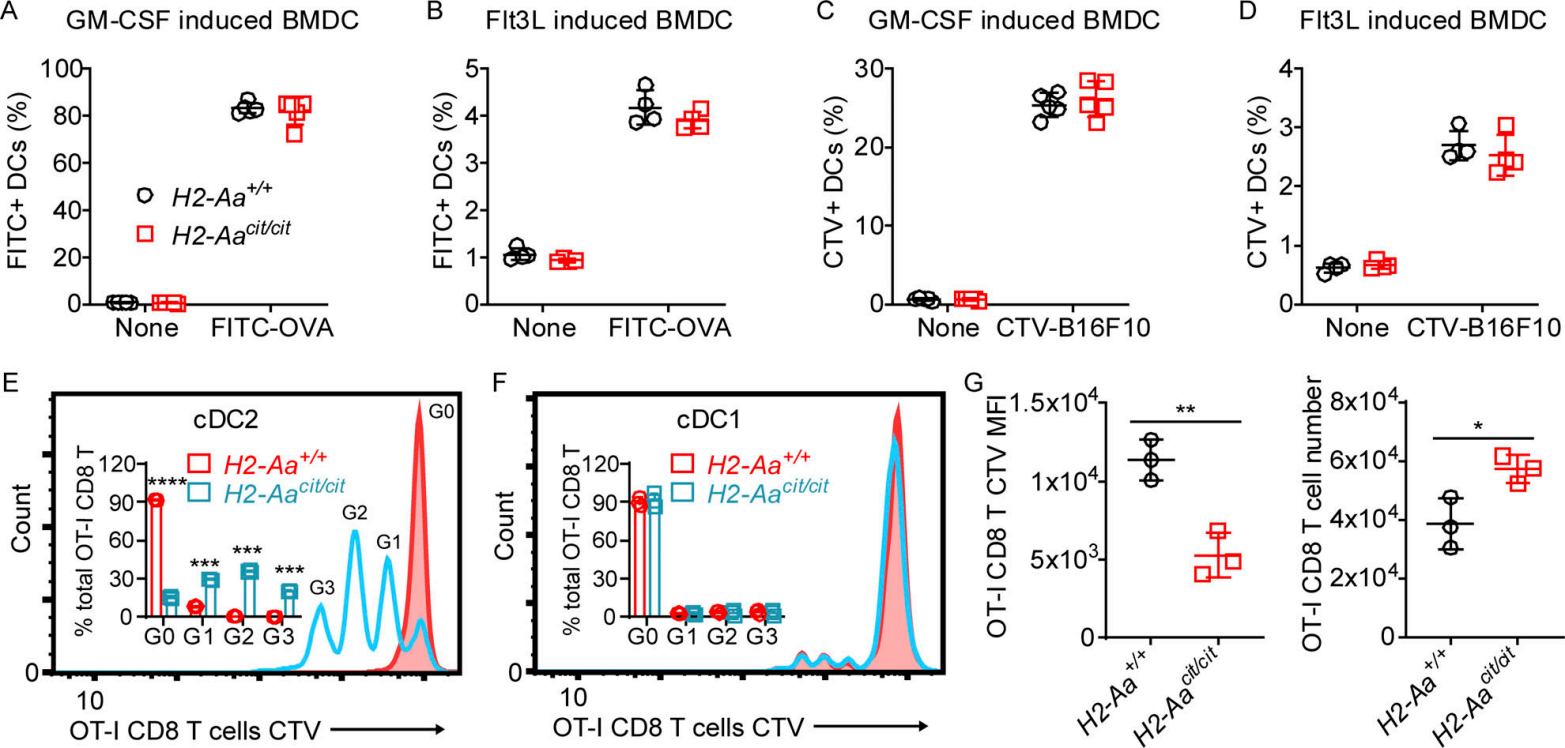
***H2-Aa***^***cit/cit***^ **cDC2 have increased cross-priming activity. (A–D)** Antigen uptake assays of BMDC (*n* = 4 or 5 per group). Frequency of FITC-positive BMDC (A and B) or CellTrace Violet (CTV)-positive BMDC (C and D) after incubation with FITC-labeled OVA (A and B) or after co-culture with CTV-labeled B16F10 cells (C and D). BMDC were induced from bone marrow cells by GM-CSF (A and C) or Flt3L (B and D). **(E and F)** In vitro cross-priming by cDC2 (E) and cDC1 (F). Representative flow cytometric histogram plots of CTV-labeled naïve OT-I CD8 T cells after co-culture (3 days) with DC purified from draining lymph nodes on day 6 after inoculation of mice with B16F10-OVA tumors. cDC2 cells were sorted as Lin− CD45^+^ Ly6C− CD11c+ Xcr1− CD11b+ and cDC1 cells were sorted as Lin− CD45^+^ Ly6C− CD11c+ Xcr1+ CD11b−. Inset, ratio of peak area for G0, G1, G2, or G3/total peak area (G0+G1+G2+G3). **(G)** In vivo cross-priming by DC. WT and *H2-Aa*^*cit/cit*^ mice were injected intravenously with equal amounts of CTV-labeled OT-I CD8 T cells, and 1 day later, recipients received OVA by i.p. injection. 4 days after OVA injection, splenocytes were collected and analyzed by flow cytometry. CTV mean fluorescence intensity (MFI, left), and the total number of OT-I CD8 T cells in the spleen (right). Three mice per group are shown (E–G). Data points represent individual mice (A–G). Data are representative of two independent experiments (A–G). WT littermates were used as controls (A–G). Error bars indicate SD (A–G). P values were determined by Student’s *t* test (A–G); no differences between genotypes were found in A–D and F. *P < 0.05; **P < 0.01; ***P < 0.001; ****P < 0.0001.

**Figure S2.**
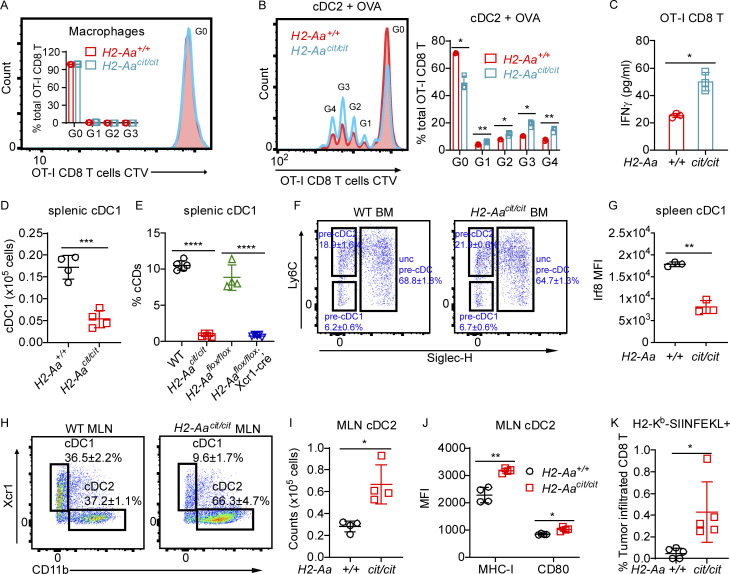
**Elevated cDC2 cross priming activity, cell counts, and MHC-I expression in *H2-Aa***^***cit/cit***^ **mice. (A)** In vitro cross-priming by macrophages. Representative flow cytometric histogram plots of CTV-labeled naïve OT-I CD8 T cells after co-culture (3 days) with macrophages purified from draining lymph nodes on day 6 after inoculation of mice with B16F10-OVA tumors. Macrophages were sorted as Lin− CD45^+^ CD11b+ F4/80+. Inset: Ratio of peak area for G0, G1, G2, or G3/total peak area (G0+G1+G2+G3). **(B and C)** In vitro cross-priming by cDC2. CTV-labeled naïve OT-I CD8 T cells were co-cultured (3 days) with OVA (100 μg/ml) and cDC2 purified from Flt3L (100 ng/ml) induced WT or *H2-Aa*^*cit/cit*^ BMDCs. **(B)** Representative flow cytometric histogram plots of CTV-labeled naïve OT-I CD8 T cells after co-culture. cDC2 were sorted as Lin− CD45^+^ Ly6C− CD11c+ CD11b^hi^ Xcr1^lo^. Right: Ratio of peak area for G0, G1, G2, G3, or G4/total peak area (G0+G1+G2+G3+G4). **(C)** ELISA analysis of IFNγ secreted by OT-I CD8 T cells after co-culture. **(D)** The total number of cDC1 in spleens of WT and *H2-Aa*^*cit/cit*^ mice. **(E)** The frequencies of splenic cDC1 in mice of the indicated genotypes. **(F)** Representative flow cytometry plots of pre-cDC in the bone marrow. Bone marrow cells were gated on Lin− CD11c+ CD172a− Flt3+. Unc pre-cDC, uncommitted pre-cDC. **(G)** Intracellular Irf8 mean fluorescence intensity (MFI) in splenic cDC1. **(H)** Representative flow cytometry plots of the indicated cDC populations in mesenteric lymph nodes (MLN) of WT and *H2-Aa*^*cit/cit*^ mice. Cells were gated on Lin− CD45^+^ Ly6C− CD11c+. **(I)** The total number of cDC2 in MLN of WT and *H2-Aa*^*cit/cit*^ mice. **(J)** MHC-I and CD80 MFI on cDC2. **(K)** Frequency of H2-K^b^-SIINFEKL tetramer positive tumor infiltrated CD8 T cells in WT or *H2-Aa*^*cit/cit*^ mice on day 9 after s.c inoculation with B16F10-OVA cells. Data points represent individual mice (A–E, G, and I–K). Data are representative of two independent experiments (A–K). WT littermates (A–D and F–K) and WT C57BL/6J (E) from JAX were used as controls. Error bars indicate SD (A–E, G, and I–K). P values were determined by Student’s *t* test (A–E, G, and I–K). *n* = 3 per group (A–C, F, and G), *n* = 4 per group (D, E, and H−K). *P < 0.05; **P < 0.01; ***P < 0.001; ****P < 0.0001.

To assess the intrinsic APC functionality of *H2-Aa*^*cit/cit*^ DC, we first analyzed antigen uptake by WT and *H2-Aa*^*cit/cit*^ GM-CSF-induced bone marrow–derived DC (BMDC) and Flt3L-induced BMDC in vitro. *H2-Aa*^*cit/cit*^ BMDC took up soluble antigen (FITC-ovalbumin [OVA], Fig. 3, A and B) and cell-associated antigen (CellTrace Violet [CTV]-labeled B16F10, Fig. 3, C and D), similar to WT BMDC. To assess whether *H2-Aa* mutation affected the capacity of DC to cross-prime CD8 T cells, we challenged *H2-Aa*^*cit/cit*^ mice with OVA-expressing B16F10 cells (B16F10-OVA) and 6 days later purified DC from draining lymph nodes for testing in vitro. The proliferation of dye-labeled *H2-Aa*^*+/+*^ naïve OT-I CD8 T cells co-cultured with the *H2-Aa*^*cit/cit*^ DC was measured as a readout of DC cross-priming ability. *H2-Aa*^*cit/cit*^ cDC2 promoted more OT-I CD8 T cell proliferation compared with *H2-Aa*^*+/+*^ cDC2, without the addition of SIINFEKL peptide (OVA epitope presented by MHC-I) to the culture medium (Fig. 3 E). In contrast, *H2-Aa*^*cit/cit*^ cDC1 (Fig. 3 F) or macrophages (Fig. S2 A) failed to do so. Enhanced cross-priming of OT-I CD8 T cells in vitro by cDC2 derived from the bone marrow of *H2-Aa*^*cit/cit*^ mice was also observed (Fig. S2 B); correspondingly, more IFNγ was detected in the culture medium from OT-I CD8 T cells co-cultured with bone marrow–derived *H2-Aa*^*cit/cit*^ cDC2 than with WT cDC2 and OVA (Fig. S2 C). To test in vivo DC cross-priming, we adoptively transferred dye-labeled *H2-Aa*^*+/+*^ OT-I CD8 T cells to either *H2-Aa*^*+/+*^ or *H2-Aa*^*cit/cit*^ mice, which were immunized with OVA. 4 days later, we found that the OT-I CD8 T cells in *H2-Aa*^*cit/cit*^ mice had proliferated more than those in *H2-Aa*^*+/+*^ mice, leading to more OT-I cells in the spleens of *H2-Aa*^*cit/cit*^ mice compared with *H2-Aa*^*+/+*^ mice (Fig. 3 G). These data indicate that the *H2-Aa*^*cit*^ mutation increased the cross-priming ability of cDC2.

### *H2-Aa*–deficient cDC2 are increased in number and in an activated state

**Figure 4.**
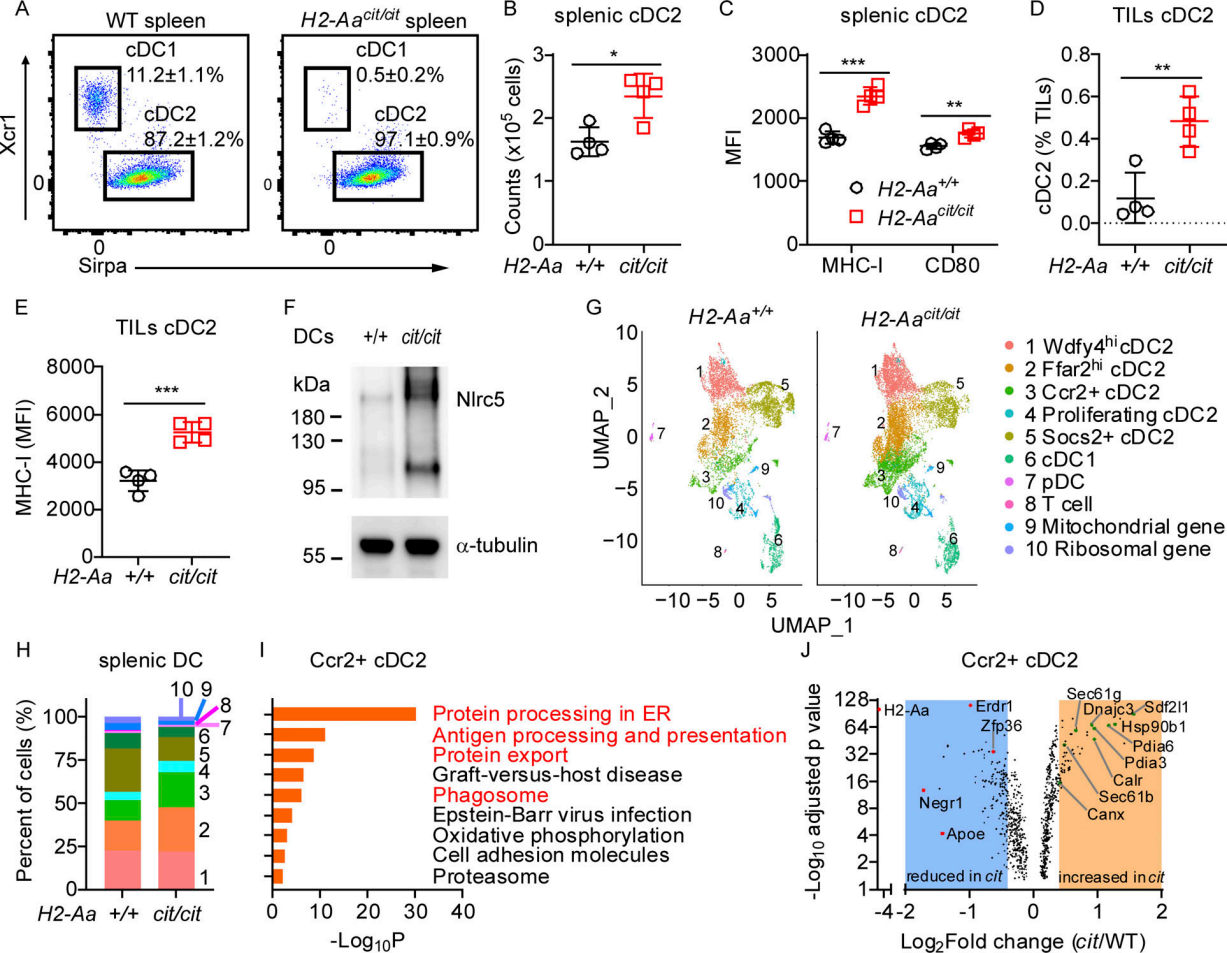
***H2-Aa* deficiency increased cDC2 and altered their transcriptional program to promote cross-presentation. (A)** Representative flow cytometry plots of the indicated cDC populations in spleens of WT and *H2-Aa*^*cit/cit*^ mice. Cells were gated on Lin− CD45^+^ Ly6C− CD11c+. **(B)** The total number of cDC2 in spleens of WT and *H2-Aa*^*cit/cit*^ mice. **(C)** MHC-I and CD80 mean fluorescence intensity (MFI) on cDC2. **(D and E)** Frequency (D) and MHC-I MFI (E) of cDC2 among tumor-infiltrating lymphocytes isolated from B16F10 melanomas collected on day 11 after B16F10 inoculation into the flank of mice. **(F)** Immunoblot analysis of Nlrc5 in lysates of panDC enriched from spleens of WT and *H2-Aa*^*cit/cit*^*(cit/cit)* mice. α-tubulin was used as a loading control. **(G)** Uniform Manifold Approximation and Projection (UMAP) clustering of scRNA-seq data from splenic DC sorted from 3 naïve *H2-Aa*^*cit/cit*^ mice (right) and 3 naïve WT littermates (left), showing 10 color-coded clusters at a resolution of 0.2. **(H)** Proportion of each cell cluster identified in G. **(I)** KEGG pathway enrichment analysis of genes significantly increased in *H2-Aa*^*cit/cit*^ relative to WT Ccr2+ cDC2 (adjusted P ≤ 0.05, *n* = 410). One-sided hypergeometric test was used to determine the statistical significance of enrichment. **(J)** Volcano plot showing differentially expressed genes in WT versus *H2-Aa*^*cit/cit*^ *(cit)* Ccr2+ cDC2 (adjusted P ≤ 0.05, *n* = 854). Shaded areas contain genes with Log_2_Fold change (FC) > 0.4 and Log_2_FC less than −0.4. Data points represent individual mice with four mice per group (B–E). Data are representative of one experiment (G–J) or two independent experiments (A–F). WT littermates were used as controls (A–H). Error bars indicate SD (B–E). P values were determined by Student’s *t* test (B–E). *P < 0.05; **P < 0.01; ***P < 0.001. Source data are available for this figure: SourceData F4.

Our data suggested that *H2-Aa*–deficient cDC2 are critical for melanoma inhibition. We found that cDC2 numbers and frequencies were increased, while cDC1 were reduced in *H2-Aa*^*cit/cit*^ mice (Fig. 4, A and B; and Fig. S2 D). The reduced ratio of cDC1 was also noticed in *H2-Aa*^*flox/flox*^;Xcr1-cre mice (Fig. S2 E). However, we found that DC precursor frequencies in the bone marrow were comparable between WT and *H2-Aa*^*cit/cit*^ mice (Fig. S2 F), suggesting that loss of *H2-Aa* affected the terminal differentiation of cDC in peripheral tissues (Mildner and Jung, 2014; Puhr et al., 2015; Sichien et al., 2017). Interferon regulatory factor 8 (IRF8) is required for stabilizing the development of pre-cDC1 into the cDC1 and is essential for cDC1 identity maintenance (Grajales-Reyes et al., 2015; Lanca et al., 2022; Sichien et al., 2016). We detected reduced IRF8 expression in *H2-Aa*^*cit/cit*^ splenic cDC1, which may contribute to the loss of cDC1 in *H2-Aa*^*cit/cit*^ mice (Fig. S2 G).

*H2-Aa*^*cit/cit*^ splenic cDC2 expressed more MHC-I and CD80 (a costimulatory molecule and marker of DC activation) on the cell surface than WT cDC2 (Fig. 4 C). Similar results were obtained for *H2-Aa*^*cit/cit*^ cDC2 in mesenteric lymph nodes (MLN) (Fig. S2, H–J). Importantly, greater numbers of cDC2 infiltrated B16F10 melanomas in *H2-Aa*^*cit/cit*^ mice than in WT mice (Fig. 4 D), and *H2-Aa*^*cit/cit*^ cDC2 within tumors expressed more MHC-I than WT cDC2 (Fig. 4 E). Notably, we found that NLRC5, a key positive transcriptional regulator of MHC class I genes (Kobayashi and van den Elsen, 2012), was more abundant in *H2-Aa*^*cit/cit*^ splenic DC than in WT DC (Fig. 4 F). Consistently, a higher proportion of SIINFEKL (OVA 257–264)-specific TCR-expressing CD8 T cells infiltrated B16F10-OVA melanoma in *H2-Aa*^*cit/cit*^ mice than in WT mice (Fig. S2 K), suggesting that tumor antigen-specific immune responses were more vigorous in *H2-Aa*^*cit/cit*^ mice. These data indicate that cDC2 in *H2-Aa*^*cit/cit*^ mice are increased in number and are qualitatively altered as well, exhibiting a more highly activated state than those in WT mice, both in spleens and tumors, correlating with enhanced cross-priming activity in *H2-Aa*^*cit/cit*^ mice.

**Figure S3.**
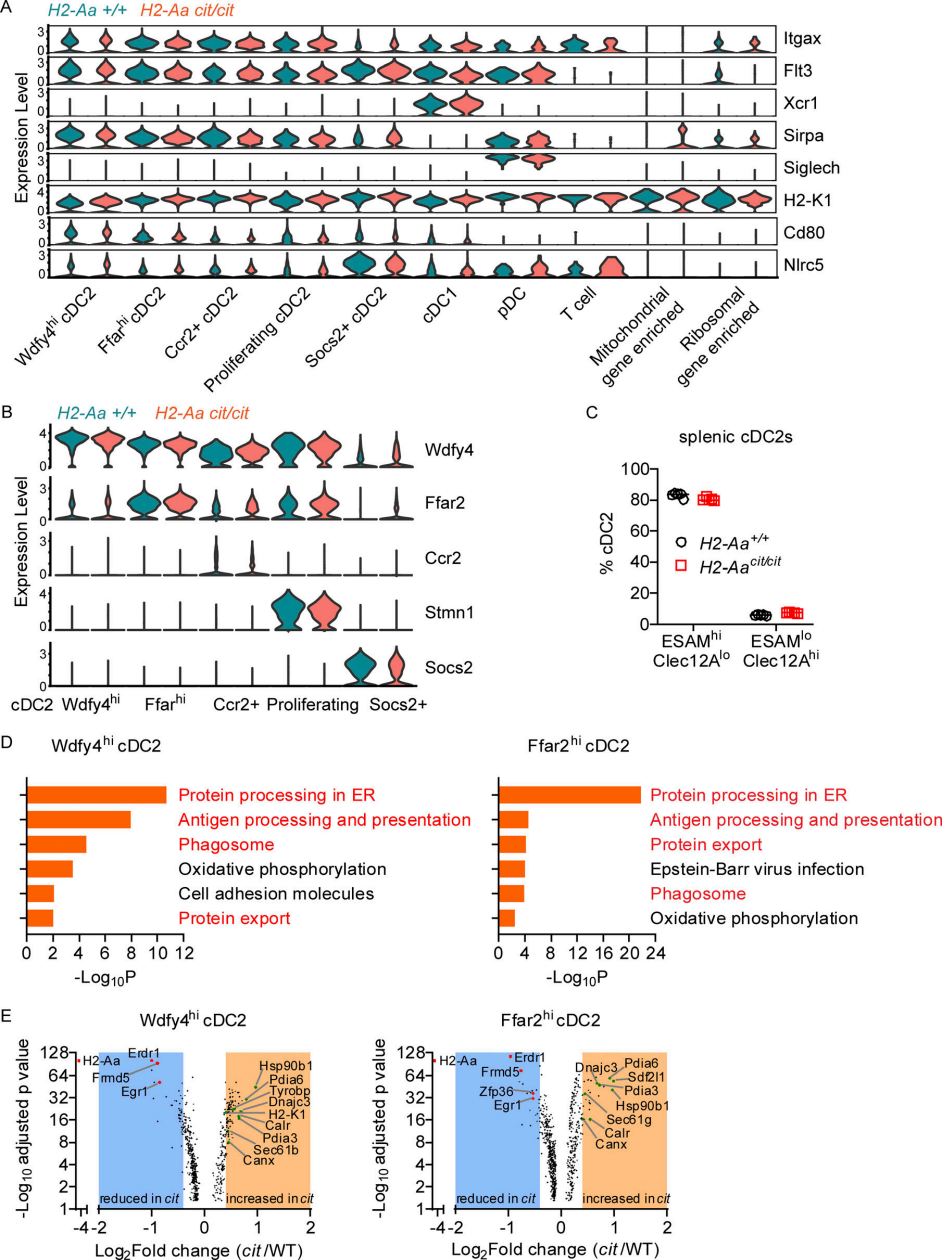
**scRNA-seq analysis of splenic DCs sorted from WT and *H2-Aa***^***cit/cit***^ **mice. (A and B)** Violin plots showing the expression distribution of DC subset-defining markers (A) or cDC2 subset-defining markers (B) in different clusters from Fig. 4 G. **(C)** Frequencies of ESAM^hi^ or Clec12A^hi^ splenic cDC2. Data points represent individual mice (*n* = 5 per group). Error bars indicate SD. No significant difference by Student’s *t* test. **(D)** KEGG pathway enrichment analysis of genes significantly increased in *H2-Aa*^*cit/cit*^ relative to WT Wdfy4^hi^ cDC2 (adjusted P ≤ 0.05, *n* = 203) and Ffar^hi^ cDC2 (adjusted P ≤ 0.05, *n* = 261). One-sided hypergeometric test was used to determine statistical significance of enrichment. **(E)** Volcano plot showing differentially expressed genes in WT versus *H2-Aa*^*cit/cit*^ *(cit)* Wdfy4^hi^ cDC2 (*n* = 570) and Ffar^hi^ cDC2 (*n* = 688). Adjusted P ≤ 0.05. Shaded areas contain genes with Log_2_Fold change (FC) >0.4 and Log_2_FC less than −0.4. Data are representative of one experiment (A, B, D, and E) or two experiments (C). WT littermates were used as controls (A–E).

To explore how MHC-II deficiency due to *H2-Aa* deletion leads to the above effects on cDC2, we employed single-cell RNA sequencing (scRNA-Seq) of splenic DC. We analyzed transcriptome data from 7,806 WT cells and 11,521 *H2-Aa*^*cit/cit*^ cells (Data S1) and verified DC identity using the canonical DC marker Itgax (CD11c) and the transcription factor Flt3, which are essential for the development and maintenance of DC in the spleen (Waskow et al., 2008). Expression levels of DC subset-defining markers, such as Xcr1, Sirpα, and Siglech (Cabeza-Cabrerizo et al., 2021; Zhang et al., 2006), were assessed to determine cDC1, cDC2, or plasmacytoid DC (pDC) identity, respectively (Fig. S3 A). Consistent with earlier findings (Schroth et al., 2023), we observed that cDC2 are diverse, forming five distinct subsets when analyzed with high-resolution clustering (Fig. 4 G). WT and *H2-Aa*^*cit/cit*^ cDC2 clusters were similarly identified by their differential expression of conserved marker genes Wdfy4, Ffar2, Ccr2, Stmn1 (proliferation marker), and Socs2 (Fig. S3 B). While there was overlap in the UMAP spaces occupied by WT and *H2-Aa*^*cit/cit*^ DC, we noted dramatic differences (Fig. 4 H). We observed an overall increased frequency of cDC2 (clusters 1–5) in the *H2-Aa*^*cit/cit*^ sample, especially Ffar2^hi^ cDC2 (cluster 2) and Ccr2+ cDC2 (cluster 3), compared with cDC2 in the WT sample (Fig. 4 H). Ccr2+ cDC2, also known as inflammatory cDC2 with upregulated IRF8 relative to their Ccr2− cDC2 counterparts, performed better than their classical cDC2 counterparts in stimulating CD8 T cell immunity (Bosteels et al., 2020). Proliferating cDC2 (cluster 4) were also increased among *H2-Aa*^*cit/cit*^ DC. This cluster expressed increased markers of cellular proliferation including Stmn1, Top2a, Mki67, and Pclaf (Data S1), which may contribute to increased immunological response and increased cell numbers. In contrast, Socs2+ cDC2 (cluster 5) were present at a lower frequency among *H2-Aa*^*cit/cit*^ DC, and such cells were reported to negatively regulate TLR-induced DC activation (Posselt et al., 2011). To explore whether the observed heterogeneity in cDC2 might be correlated with the previously reported dichotomy of cDC2 into ESAM^hi^ and Clec12A^hi^ populations in the murine spleen (Lewis et al., 2011; Amon et al., 2024), we analyzed ESAM and Clec12A expression on splenic cDC2. Flow cytometry results indicated that the expression levels of ESAM and Clec12A varied significantly among splenic cDC2. However, there were no substantial differences in the frequencies of ESAM^hi^ cDC2 or Clec12A^hi^ cDC2 between WT and *H2-Aa*^*cit/cit*^ cDC2 (Fig. S3 C).

Kyoto Encyclopedia of Genes and Genomes (KEGG) pathway enrichment analysis of genes with elevated expression in *H2-Aa*^*cit/cit*^ cDC2 revealed enrichment of genes involved in protein processing in the ER, antigen processing and presentation, and protein export, and phagosome-associated genes (Fig. 4 I and Fig. S3 D). These pathways are involved in antigen presentation, especially cross-presentation. Among the genes with enhanced expression in *H2-Aa*^*cit/cit*^ cDC2 compared with WT cDC2 (Fig. 4 J, Fig. S3 E, and Data S2) were Pdia3 (also named Erp57), Canx (calnexin), and Calr (calreticulin), which load peptides onto MHC-I molecules (Pishesha et al., 2022); Sec61 and Hsp90b1 (also named GRP94) are required for antigen export from phagosomes to the cytosol and for cross-presentation (Joffre et al., 2012); Sdf2l1 and Dnajc3 (also known as p58IPK, Erdj6) promote ER homeostasis by preventing misfolded protein aggregation and thus improve DC immunobiology (Hanafusa et al., 2019; Rutkowski et al., 2007; Salvagno and Cubillos-Ruiz, 2019); and Pdia6, which is essential for lymphoid and myeloid development (Choi et al., 2020). By contrast, genes depressed in *H2-Aa*^*cit/cit*^ cDC2 compared with WT cDC2 (Fig. 4 J), such as Negr1 and Apoe, inhibit the IL-6 and IL-1β pathways, respectively (Sauter et al., 2021; Yoo and Lee, 2023). Erdr1 and Zfp36 (also known as TPP), which showed increased expression in different cDC2 clusters in WT as compared with *H2-Aa*^*cit/cit*^ cells (Fig. 4 J and Fig. S3 E), are concerned with cell apoptosis and inhibited DC maturation, respectively (Emmons et al., 2008; Soto et al., 2017). Overall, the data suggest causative explanations for the increased cDC2 population, elevated MHC-I expression, and enhanced cross-priming activity in *H2-Aa*^*cit/cit*^ mice.

### Cell autonomous regulation of cDC function by MHC-II

**Figure 5.**
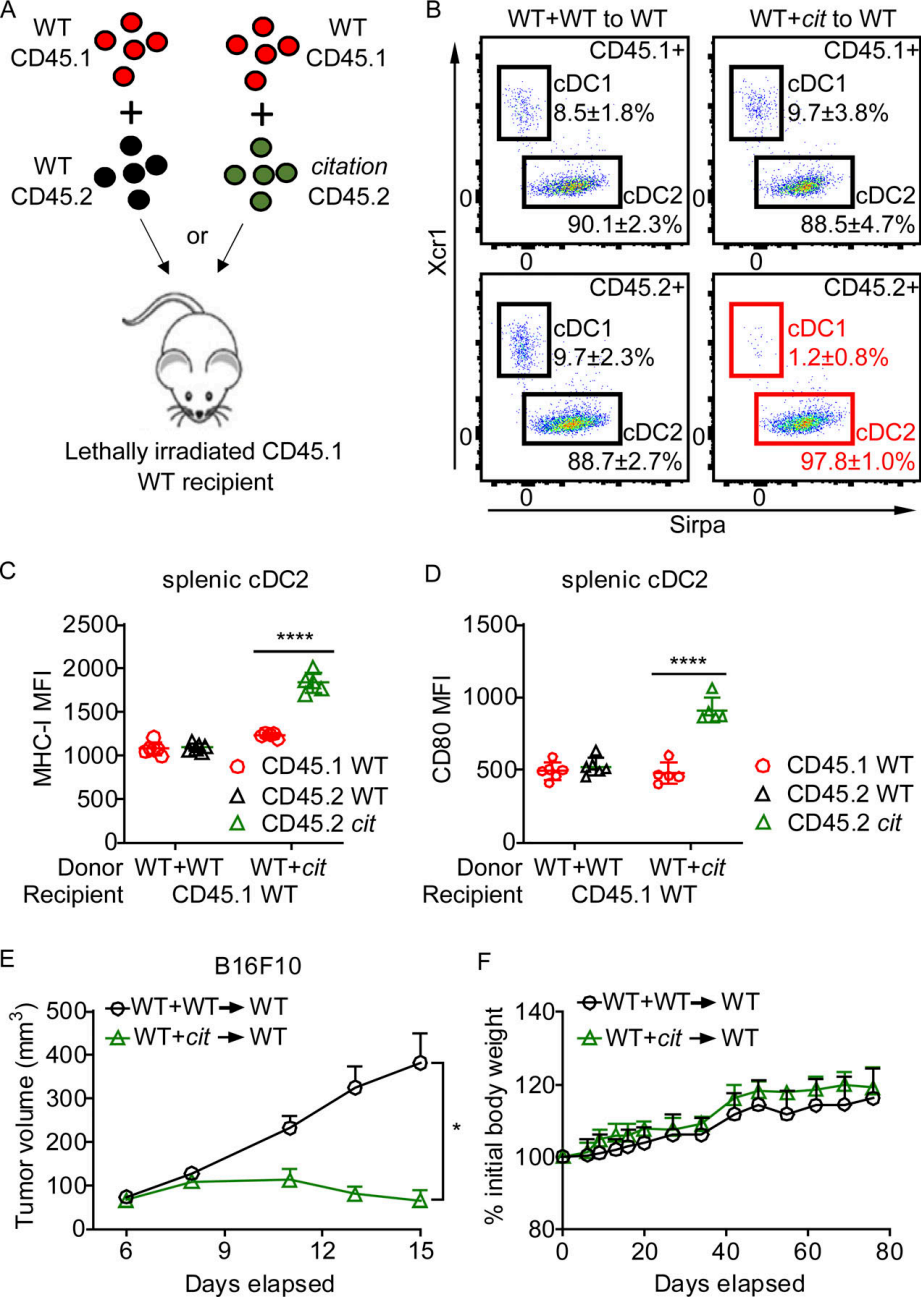
**Cell-autonomous regulation of cDC function by MHC-II. (A)** Diagram of mixed bone marrow transplantation. A 1:1 mixture of *H2-Aa*^*cit/cit*^ (CD45.2) or WT (CD45.2) bone marrow cells and congenic WT (CD45.1) bone marrow cells were transferred to lethally irradiated WT (CD45.1) recipients. **(B)** Representative flow cytometry plots of the indicated cDC populations in spleens of bone marrow chimeric mice. Reduced cDC1 and increased cDC2 frequencies were observed among cDC derived from CD45.2+ *H2-Aa*^*cit/cit*^ BM (highlighted in red). **(C and D)** Expression of MHC-I (C) and CD80 (D) on splenic cDC2 from bone marrow chimeric mice. MFI, mean fluorescence intensity. **(E)** Tumor growth curve of B16F10 melanoma after s.c. inoculation on day 0 into the flank of bone marrow chimeric mice 12 wk after bone marrow transplantation. No PD1 antibody was administered. **(F)** Body weight relative to weight on the day of bone marrow transplantation (day 0). Data points represent individual mice (C and D). Data are representative of two independent experiments (B–F). WT littermates were used as bone marrow donor controls (B–F). Error bars indicate SD (C, D, and F) or SEM (E). P values were determined by Student’s *t* test (C and D) or two-way ANOVA with post-hoc Tukey test (E). *n* = 5 or 6 recipients per group (B–F). *P < 0.05; ****P < 0.0001.

**Figure S4.**
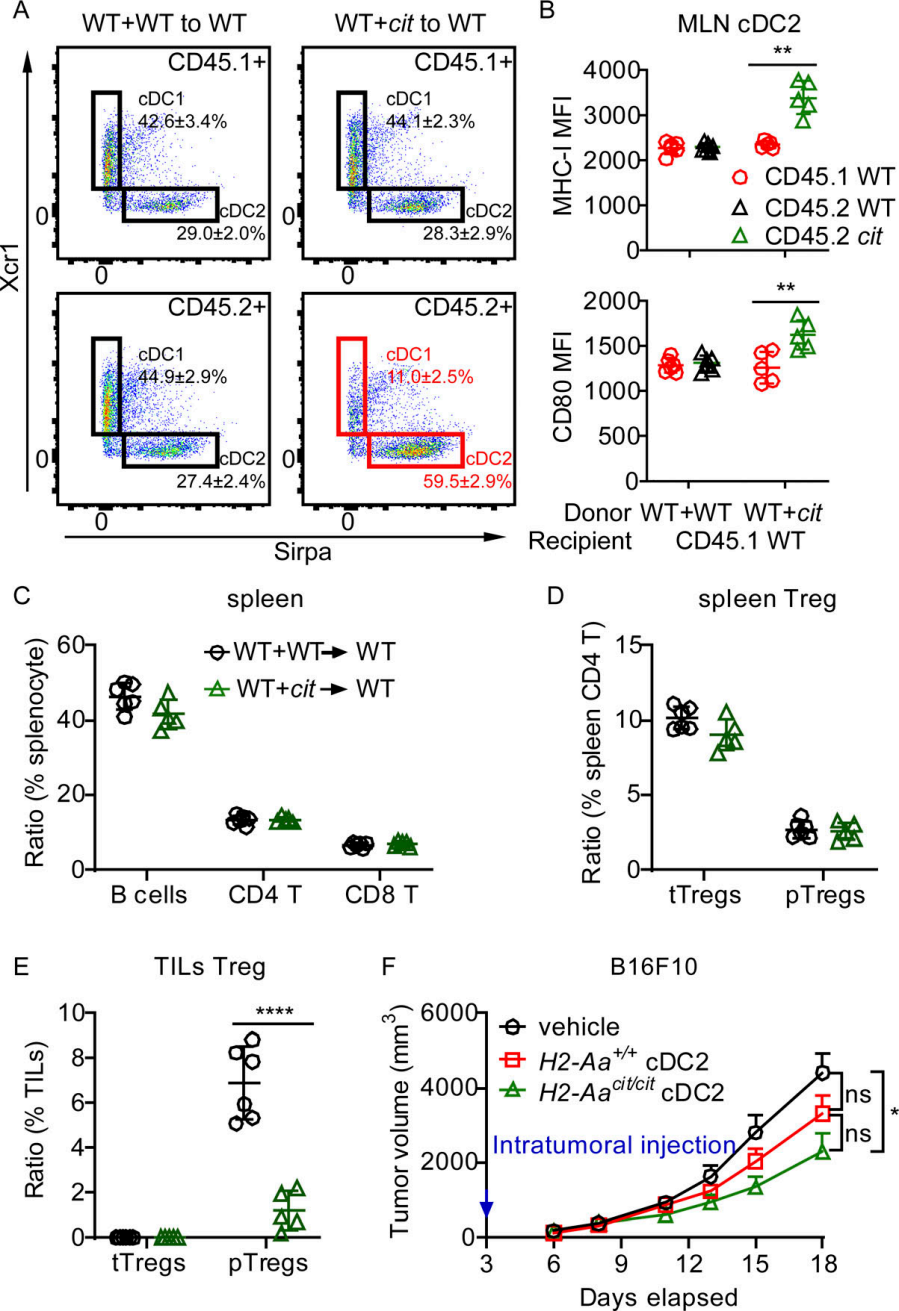
**Lymphocyte frequencies in mixed bone marrow chimeric mice. (A–E)** A 1:1 mixture of *H2-Aa*^*cit/cit*^ (*cit*, CD45.2) or WT (CD45.2) bone marrow cells and congenic WT (CD45.1) bone marrow cells were transferred to lethally irradiated WT (CD45.1) recipients (*n* = 5 or 6 recipients per group). **(A)** Representative flow cytometry plots of the indicated cDC populations in mesenteric lymph nodes (MLN) of bone marrow chimeric mice. Reduced cDC1 and increased cDC2 frequencies were observed among cDC derived from CD45.2 *H2-Aa*^*cit/cit*^ BM (*cit*, highlighted in red). **(B)** Expression of MHC-I (upper panel) and CD80 (lower panel) on MLN cDC2 from bone marrow chimeric mice. MFI, mean fluorescence intensity. **(C and D)** Frequencies of B cells, CD4 T cells, CD8 T cells (C), and Treg (D) in the spleens of bone marrow chimeric mice. **(E)** Frequency of Treg in tumor infiltrating lymphocytes from B16F10 melanoma collected on day 21 after B16F10 inoculation in the flank of bone marrow chimeric mice. **(F)** Tumor growth curves of B16F10 melanoma after s.c. inoculation on day 0 into the flank of WT mice. Naïve cDC2 cells were intratumorally injected on day 3 (*n* = 6–8 per group). Data points represent individual mice (B–E). Data are representative of two independent experiments (A–F). Error bars indicate SD (B–E) or SEM (F). P values were determined by Student’s *t* test (B–E) or two-way ANOVA with post-hoc Tukey test (F). *P < 0.05; **P < 0.01; ****P < 0.0001; ns, not significant.

To assess whether the numerical and functional enhancements of cDC2 in *H2-Aa*^*cit/cit*^ mice were due to cell intrinsic *H2-Aa* deficiency, mixed bone marrow chimeras were established by transferring a 1:1 mixture of WT (CD45.1) BM and BM from either *H2-Aa*^*cit/cit*^ (CD45.2) or WT (CD45.2) mice into lethally irradiated WT (CD45.1) recipients (Fig. 5 A). 12 wk after reconstitution, reduced cDC1 and increased cDC2 populations were observed among cDC derived from *H2-Aa*^*cit/cit*^ BM, whereas they were not observed in cDC derived from WT BM (Fig. 5 B and Fig. S4 A). Furthermore, elevated expression of MHC-I and CD80 was only observed on cDC2 derived from *H2-Aa*^*cit/cit*^ BM (Fig. 5, C and D; and Fig. S4 B). Importantly, recipients of *H2-Aa*^*cit/cit*^ BM (mixed 1:1 with CD45.1 WT BM) could still strongly inhibit B16F10 melanoma (Fig. 5 E), while they had similar numbers of splenic B cells and both CD4 and CD8 T cells as WT BM recipients (Fig. S4 C). Treg, including tTreg and pTreg, were present at similar frequencies in recipients of *H2-Aa*^*cit/cit*^ + WT BM and in recipients of WT + WT BM (Fig. S4 D), whereas fewer pTreg were recruited to tumor microenvironment in recipients of *H2-Aa*^*cit/cit*^ + WT BM than in recipients of WT + WT BM (Fig. S4 E). The body weights remained comparable between recipients of *H2-Aa*^*cit/cit*^ + WT BM and recipients of WT + WT BM, indicating that hosts with *H2-Aa*^*cit/cit*^ BM did not develop autoimmunity (Fig. 5 F). These results clearly demonstrate the existence of cell-autonomous regulation of cDC numbers and function by MHC-II. Insofar as the extreme CD4 T cell deficiency characteristic of *cit* homozygotes was not observed in chimeras with WT thymic epithelium while the tumor resistance was still observed, we conclude that CD4 T cell deficiency (including Treg deficiency) is not a prerequisite for restriction of tumor growth in *H2-Aa*^*cit/cit*^ mice. However, we note that productive Treg-cDC interactions are presumably abrogated in these mice. Intratumoral injection of pure *H2-Aa*^*cit/cit*^ cDC2 into B16F10 melanoma also inhibited tumor growth in WT mice (Fig. S4 F).

### Loss of Treg facilitates tumor inhibition in *H2-Aa*^*cit/cit*^ mice

**Figure 6.**
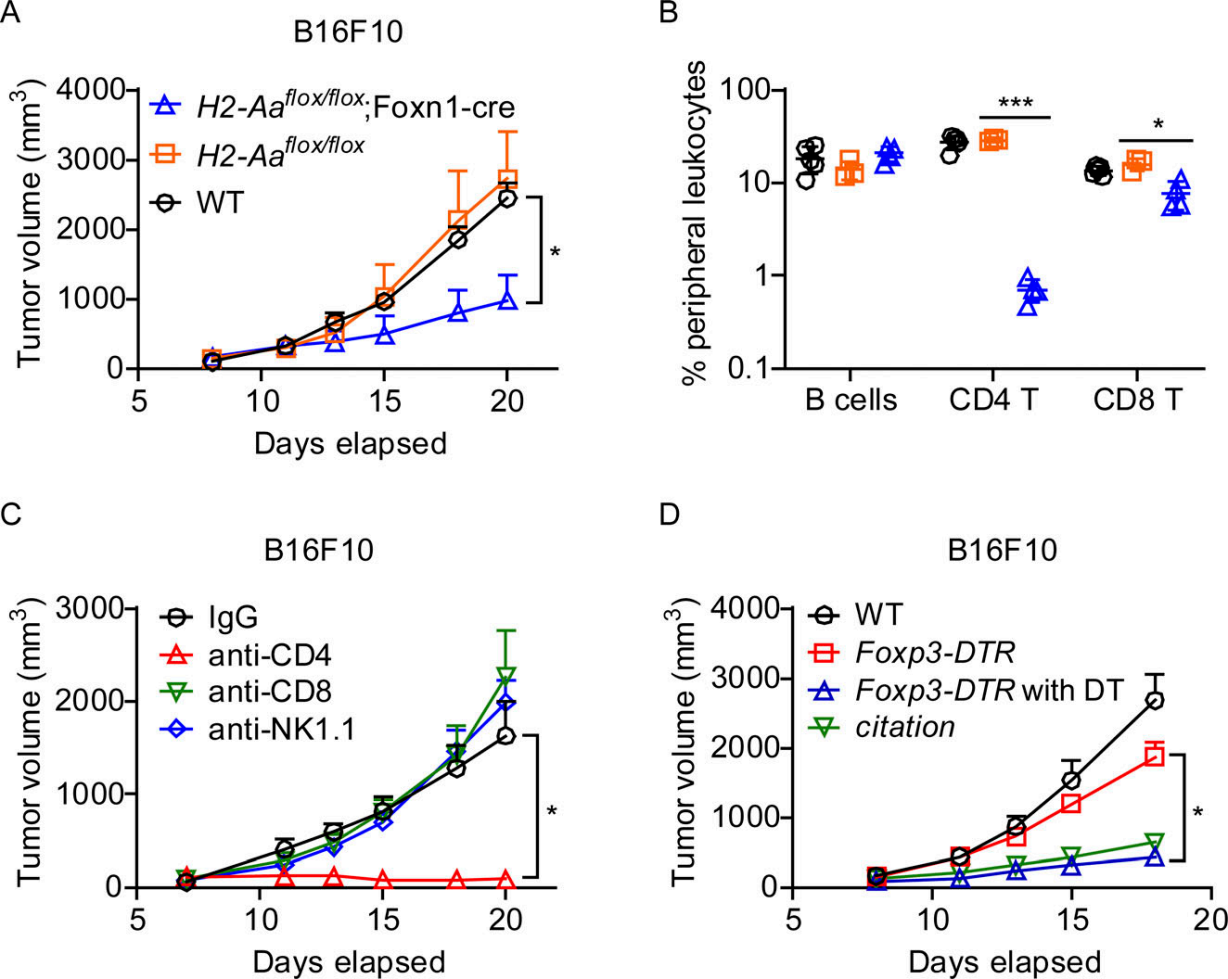
**Treg depletion inhibited melanoma growth.** B16F10 cells were injected s.c. on day 0 into the flank of mice. No PD1 antibody was administered. **(A and B)** Tumor growth curve (A) and frequencies of B cells, CD4 T cells, and CD8 T cells in the peripheral blood (B) on day 20 (*n* = 3–5 per group). **(C)** Tumor growth curve in the presence of cell depleting antibodies. Anti-CD4, anti-CD8, anti-NK1.1, or control IgG were injected i.p. into WT mice on days 0, 3, 6, 9, 12, and 15 after tumor inoculation to deplete the corresponding cells (*n* = 5 per group). **(D)** Tumor growth curve in *Foxp3-DTR* mice. 1 μg diphtheria toxin (DT) per mouse was injected i.p. on a daily basis for five consecutive days (day −5 to −1) to deplete Treg (Foxp3+ cells) in *Foxp3-DTR* mice (*Foxp3-DTR* with DT) (*n* = 4–8 per group). Data points represent individual mice in B. Data are representative of two independent experiments (A–D). WT C57BL/6J mice from JAX were used as controls (A–D). Error bars indicate SD (B) or SEM (A, C, and D). P values were determined by two-way ANOVA with post-hoc Tukey test (A–D). *P < 0.05; ***P < 0.001.

Treg are essential for self-tolerance, but they can also impair anticancer immunity, undermining tumor surveillance and promoting tumor growth and progression (Togashi et al., 2019). To address whether lack of CD4 T cells also contributes to melanoma growth restriction in *H2-Aa*^*cit/cit*^ mice, we generated *H2-Aa*^*flox/flox*^;Foxn1-cre mice, in which *H2-Aa* was deleted specifically in thymic epithelial cells, thus blocking CD4 T cell development. We found that *H2-Aa*^*flox/flox*^;Foxn1-cre mice efficiently inhibited B16F10 melanoma growth (Fig. 6 A). The mice had very few CD4 T cells and relatively normal numbers of B cells in the peripheral blood (Fig. 6 B), suggesting that *H2-Aa* KO in thymic epithelial cells was complete and there was no leakage of Cre expression to B cells. Furthermore, strong suppression of melanoma growth was observed in WT mice with antibody-mediated CD4 T cell depletion in vivo (Fig. 6 C). Foxp3-DTR-KI mice (Kim et al., 2007) treated with diphtheria toxin also suppressed B16F10 melanoma growth (Fig. 6 D), indicating that selective Treg depletion mimicked total CD4 T cell depletion effects. These data further validated the immune suppressive role of Treg during tumor development. Together with the diminished Treg infiltration into tumors in *H2-Aa*^*cit/cit*^ mice (Fig. 1 L), the results suggest that the lack of Treg may contributes to the elite control of melanoma growth in *H2-Aa*^*cit/cit*^ mice.

### Acute MHC-II inhibition synergizes with checkpoint inhibition to suppress established melanoma

**Figure 7.**
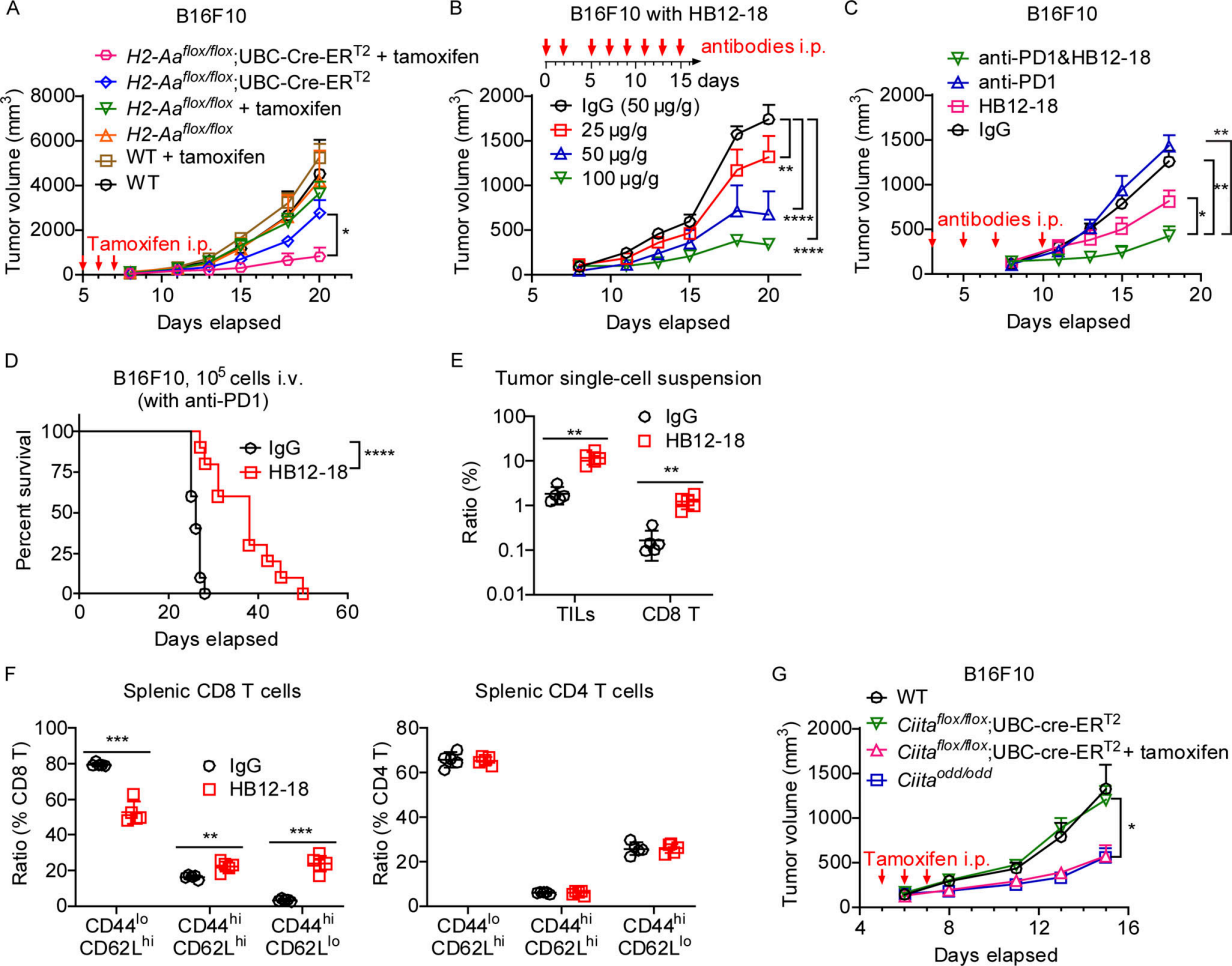
**Acute deletion of *H2-Aa* or *Ciita* or antibody-mediated blockade of MHC-II inhibited B16F10 melanoma growth. (A–C)** Tumor growth curves of B16F10 melanoma after s.c. inoculation on day 0 into the flank of mice. **(A)** Effect on tumor growth of tamoxifen-induced deletion of *H2-Aa* in the host. 100 mg tamoxifen/kg body weight was injected i.p. daily on days 5–7 (*n* = 4 per group). **(B)** Effect on tumor growth of monoclonal antibody (HB12-18) against MHC-II. Different concentrations of HB12-18 were injected i.p. into WT mice on the indicated days (*n* = 5 per group). **(C)** Effect on tumor growth of combined anti-PD1 (10 μg/g body weight) and HB12-18 (25 μg/g body weight) treatment. Antibodies were injected i.p. on days 3, 5, 7, and 10 into WT mice (*n* = 5 per group). **(D)** Survival curves of mice after i.v. inoculation with B16F10 melanoma on day 0. WT mice were intraperitoneally (i.p.) injected with HB12-18 (50 μg/g body weight) and anti-PD1 twice per week till the end of the experiment (death or euthanasia) (*n* = 10 per group). **(E)** Frequency of tumor infiltrating lymphocytes and CD8 T cells in B16F10 tumors collected on day 11 after B16F10 inoculation. Control IgG or HB12-18 monoclonal antibodies were injected i.p. into WT mice on days 3, 5, 7, and 10 (*n* = 5 per group). **(F)** WT mice were s.c inoculated with B16F10 on day 0 and i.p injected with control IgG or HB12-18 on days 3, 5, 7, and 10. On day 11, splenic CD8 T cell and CD4 T cell activation were analyzed by flow cytometry (*n* = 5 per group). **(G)** Tumor growth curves of B16F10 melanoma after s.c. inoculation on day 0 into the flank of mice. 100 mg tamoxifen/kg body weight was injected i.p. daily on days 5–7 (*n* = 7–15 per group). Data are representative of two independent experiments (A–G). WT C57BL/6J mice from JAX were used as controls (A and G). Error bars indicated SEM (A–C and G) or SD (E and F). P values were determined by two-way ANOVA with post-hoc Tukey test (A–C and G), Student’s *t* test (E and F), or log-rank test (D). Data points represent individual mice (E and F). *P < 0.05; **P < 0.01; ***P < 0.001; ****P < 0.0001.

**Figure S5.**
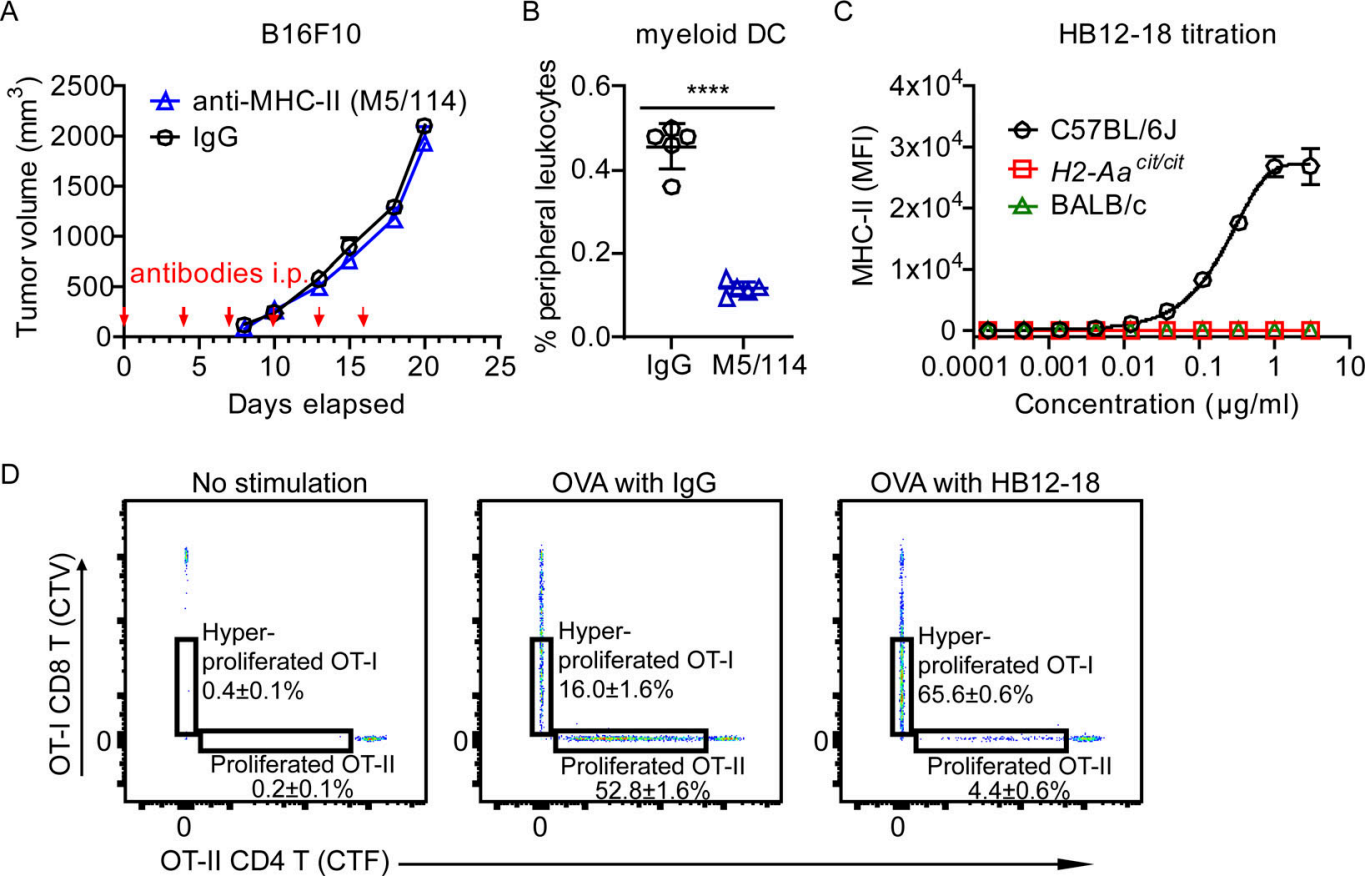
**Analysis of the MHC-II monoclonal antibody HB12-18. (A and B)** Anti-MHC-II (M5/114, 25 μg/g body weight) or control IgG were injected i.p. into WT mice on days 0, 4, 7, 10, 13, and 16 after B16F10 inoculation on day 0 into the flank of mice (*n* = 5 per group). **(A)** Tumor growth curve. **(B)** Frequencies of myeloid DC (CD11b+CD11c+) in the peripheral blood on day 15. **(C)** Splenocytes from C57BL/6J, *H2-Aa*^*cit/cit*^, or BALB/cJ mice were incubated with different concentrations of HB12-18 (*n* = 3 per group). MHC-II expression (HB12-18 reactivity) on CD19^+^ B cells was determined by flow cytometry. MFI, mean fluorescence intensity. **(D)** WT (CD45.1) mice were injected i.v. with CellTrace Violet (CTV)-labeled OT-I CD8 T cells (CD45.2) and CellTrace Far Red (CTF)-labeled OT-II CD4 T cells (CD45.2), and 1 day later, recipients were mock injected (left panel), or injected i.p. with OVA+IgG (middle panel) or OVA+HB12-18 (200 μg/mouse, right panel) (*n* = 3 per group). Representative flow cytometry plots of CTV-positive OT-I CD8 T cells (CD45.2) and CTF-positive OT-II CD4 T cells (CD45.2) in the spleens of WT recipients (CD45.1) 4 days after immunization. Data points represent individual mice (B). Data are representative of two independent experiments (A–D). Error bars indicate SD (B and C) or SEM (A). P values were determined by Student’s *t* test (B) or two-way ANOVA with post-hoc Tukey test (A); no difference between treatments was found in A. ****P < 0.0001.

We showed that melanoma growth was inhibited in DC-specific *H2-Aa* KO mice (Fig. 2 C). We further verified that acutely induced deletion of *H2-Aa* in *H2-Aa*^*flox/flox*^;UBC-cre-ER^T2^ mice treated with tamoxifen significantly suppressed the growth of preestablished melanoma (Fig. 7 A), as would be found in human patients. Considering that mice reconstituted with mixed 1:1 WT:*cit* bone marrow were also capable of strongly resisting melanoma growth (Fig. 2 A and Fig. 5 E), we decided to develop approach(es) to block MHC-II in tumor-bearing hosts, aiming to enhance their ability to reject tumors. As MHC-II is a cell surface complex, we hypothesized that blocking it with antibodies might mimic the loss of MHC-II. First, we tested a commercially available monoclonal antibody (clone M5/114) for MHC-II. However, the results were negative (Fig. S5 A), perhaps because the antibody induced strong DC depletion in vivo (Fig. S5 B). Then, we developed our own monoclonal antibodies against MHC-II. After screening >20,000 hybridomas, several monoclonal antibodies were found to specifically recognize MHC-II, block DC priming of OT-II CD4 T cells, and enhance DC cross-priming of OT-I CD8 T cells in vivo. HB12-18 was one of the positive clones (Fig. S5, C and D). We modified the Fc region of HB12-18 to eliminate its ability to fix complement or bind to Fc receptors (Kang and Jung, 2019; Schlothauer et al., 2016), purified it on a large scale, and found that it inhibited B16F10 melanoma growth in vivo in a dose-dependent manner (Fig. 7 B). Furthermore, at a relatively low dose, it could synergize with anti-PD1 to suppress melanoma growth although anti-PD1 alone was without effect (Fig. 7 C). HB12-18 treatment was also effective in a metastatic model, in which it significantly extended the survival of C57BL/6J mice inoculated i.v. with B16F10 (Fig. 7 D). The therapeutic effects were verified by results showing increased infiltration of TILs and CD8 T cells into B16F10 melanoma following HB12-18 treatment (Fig. 7 E). HB12-18 treatment significantly enhanced CD8 T cell activation while having no effect on CD4 T cells (Fig. 7 F).

While we were developing monoclonal antibodies for MHC-II, we identified a *Ciita* mutant allele which we named *oddball* (*odd*). *Ciita*^*odd/odd*^ mice dramatically constrained the growth of subcutaneously injected B16F10 tumor cells, recapitulating the effect of the *cit* mutation (Fig. 7 G). CIITA (MHC-II transactivator) serves as a master regulator of polymorphic MHC-II genes and accessory genes including the MHC-II invariant chain (Ii). CIITA controls both the constitutive and the inducible expression of MHC-II genes (Forlani et al., 2023). We then constructed *Ciita*^*flox/flox*^;UBC-cre-ER^T2^ mice. Tamoxifen-induced deletion of *Ciita* inhibited the growth of preestablished B16F10 melanomas as strongly as observed in *oddball* mice (Fig. 7 G). Taken together, the results suggest that antibodies blocking MHC-II and/or small molecular drugs inhibiting CIITA may be effective immunotherapeutic in patients with certain cancers and might also increase the efficacy of checkpoint inhibitors.

## Discussion

**Figure 8.**
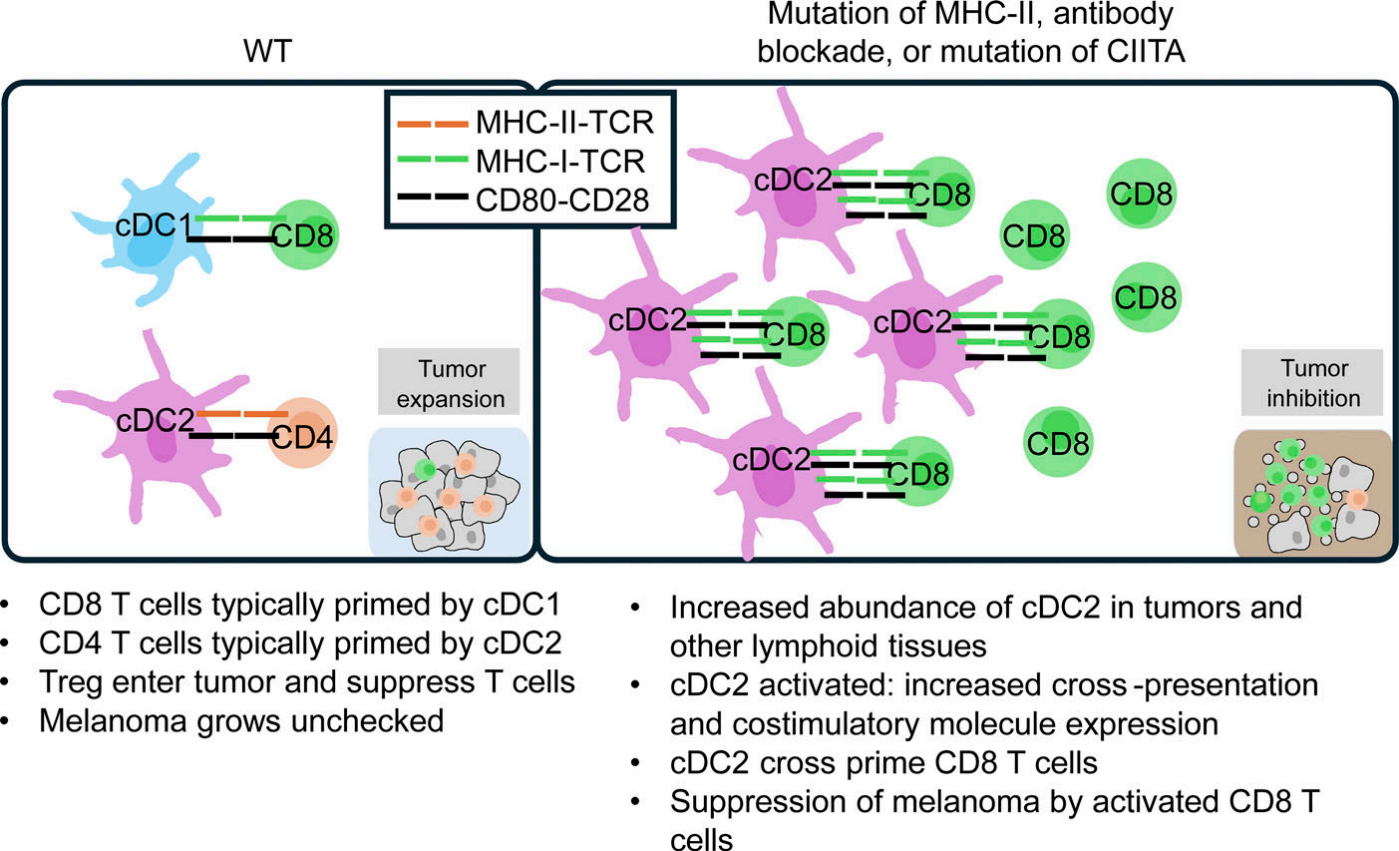
**Model of cDC2 cross-presentation regulated by MHC-II deficiency.** T cell activation requires engagement of both T cell receptors (TCR) and receptors for costimulatory molecules (e.g., CD28) by, respectively, MHC-peptide complex (p-MHC-I for CD8 T and p-MHC-II for CD4 T and Treg) and costimulatory molecules on DC (e.g., CD80). In WT mice, CD8 T cells (that mediate tumor killing) are typically primed by cDC1 and CD4 T cells (including Treg) are primed by cDC2; the outcome is that WT hosts are usually tolerant to syngeneic tumors and the tumors expand (left). In contrast, reducing or deleting MHC-II expression or function on cDC2 results in the expansion of the cDC2 population, their elevated MHC-I expression, and enhanced cDC2 cross-priming activity, all of which favor CD8 T proliferation. Conversely, the activation and proliferation of Treg are impeded due to the absence of Treg–cDC2 interaction mediated by TCR and p-MHC-II. The net result is tumor growth inhibition (right). MHC-II deficiency could be achieved by monoclonal antibody HB12-18 treatment or CIITA inhibition.

Combining random germline mutagenesis with AMM, we identified targets for highly efficacious cancer immunotherapy. Here, we described how a host *H2-Aa* mutation facilitated inhibition of melanoma growth. Our data suggested a model in which *H2-Aa* deletion upregulates both cDC2 number and cross-priming activity, leading to enhanced CD8 T cell activation and cytotoxic function as well as Treg scarcity (Fig. 8). One manifestation of these effects is elite control of tumor growth. Specifically, *H2-Aa*^*cit/cit*^ mice with melanoma exhibit a higher tumor infiltration of CD8 T cells and a lower presence of Treg compared with WT mice, consistent with a previous report (Chaoul et al., 2017). Tumor control is demonstrably enhanced by anti-PD1 antibodies, which by themselves show no effect against B16F10 melanoma. Not only checkpoint inhibition (ineffective in the majority of human cancer patients) but also CAR-T and conventional CD8 T cell therapies may be enhanced by class II HLA blockade.

Our findings are in agreement with previous work showing that MHC-II–deficient DC exhibited an activated (i.e., mature) phenotype characterized by upregulated costimulatory molecules including CD80, CD40, and CD70, which correlated with the acquisition of priming ability and loss of a tolerogenic state toward CD8 T cells (Loschko et al., 2016; Muth et al., 2012). This DC activation was intrinsic to MHC-II–deficient DC since it was not observed in WT DC present in the same animal in a mixed bone marrow chimera (Muth et al., 2012), as we also observed. A large body of work has shown that DC activation and loss of tolerogenic function are a consequence of a lack of MHC-II–mediated interactions with Treg (Binnewies et al., 2019; Muth et al., 2012; Poitrasson-Riviere et al., 2008; Schildknecht et al., 2010). Activated MHC-II–deficient DC, in turn, drive the activation of CD8 T cells (Loschko et al., 2016; Wohn et al., 2020; Zhou et al., 2023), which results in lethal CD8 T cell–mediated autoimmunity in both conditional mutants with DC-specific MHC-II deletion and in mixed bone marrow chimeras that received *MHC-II*^*−/−*^ and WT bone marrow (Muth et al., 2012; Zhou et al., 2023). In addition, pancreas and intestinal inflammation have been documented in MHC-II germline mutant mice (Mombaerts et al., 1993; Vallance et al., 1998). We did not observed weight loss, a common sign of autoimmune disease, in WT+*H2-Aa*^*cit/cit*^ mixed bone marrow chimeras for up to 11 wk after bone marrow transplantation, nor in *H2-Aa*^*flox/flox*^;Cd11c-cre mice with DC-specific *H2-Aa* deletion, nor in germline *H2-Aa*^*cit/cit*^ mice. Histological analysis of tissues or monitoring mice at older ages may reveal that autoimmune phenotypes do exist in these mice. These mice were maintained in specific pathogen-free (SPF) environments, characterized by reduced exposure to environmental antigens (Beura et al., 2016). It has yet to be determined whether subjecting *H2-Aa*^*cit/cit*^ mice to environments with higher microbial exposure would enhance CD8 T cell activity, potentially leading to autoimmune tissue damage.

CD8 T cells that develop in the context of MHC-II deficiency have been reported to express an altered TCR repertoire that includes MHC-I and -II dual-specific CD8 T cells that escape negative selection on MHC-II (Cosgrove et al., 1991; Logunova et al., 2005; Zhou et al., 2023). Such CD8 T cells are capable of mediating both autoimmunity and killing tumor cells in an MHC-II–dependent manner (Zhou et al., 2023). However, we have demonstrated that either antibodies against surface MHC-II or acute deletion of *H2-Aa* or *Ciita* produced a rapid inhibitory effect on tumor growth, recapitulating the effect of the *cit* mutation. In addition, *H2-Aa*^*cit/cit*^ mice effectively inhibited B16F10 with *H2-Aa* KO. These findings suggest that CD8 T cells that develop normally in the presence of intact MHC-II expression contain among them TCRs reactive against B16F10 melanoma. However, these CD8 T cells do not typically suppress or eradicate B16F10 melanoma in WT mice.

Cross-presentation and cross-priming are generally thought to be carried out preferentially by cDC1, whereas cDC2 are known as APCs serving CD4 T lymphocytes both in the setting of infection and cancer (Binnewies et al., 2019; Durai and Murphy, 2016). Surprisingly, we showed that deletion of *H2-Aa* specifically in cDC1 failed to recapitulate the melanoma suppression phenotype observed in *H2-Aa*^*cit/cit*^ mice, leading us to conclude that *H2-Aa*–deficient cDC2 were responsible for the suppression. This conclusion is supported by several pieces of evidence. First, cDC2 were numerically increased while cDC1 were reduced in *H2-Aa*^*cit/cit*^ lymphoid organs compared with WT lymphoid organs. Second, more *H2-Aa*^*cit/cit*^ cDC2 than WT cDC2 were recruited into tumors, and these *H2-Aa*^*cit/cit*^ cDC2 were more activated than WT cDC2, as indicated by MHC-I and CD80 expression levels. Third, scRNA-seq results showed that genes promoting DC activation and antigen cross-presentation were significantly enriched in *H2-Aa*^*cit/cit*^ cDC2 relative to WT cDC2, consistent with the elevated cross-priming activity of *H2-Aa*^*cit/cit*^ cDC2 that we measured in vitro and in vivo. These findings reveal that whereas cDC1 may be primary mediators of tumor antigen presentation in WT mice (Gardner et al., 2020), cDC2 are capable of presenting tumor antigens as well and become more effective than cDC1 in this function in the absence of MHC-II. This is consistent with previous work showing that cDC2 are capable of efficiently cross-presenting antigens to CD8 T lymphocytes in other experimental settings (Ballesteros-Tato et al., 2010; Ji et al., 2013; Sheng et al., 2017; Si et al., 2023; Theisen et al., 2018).

The signals emanating from MHC-II that prevent cDC2 activation, including limiting cell surface MHC-I and suppressing cross-presentation in a cell-intrinsic manner remain unclear. Our scRNA-seq analysis suggests that such signals support a broad transcriptional program in cDC2, and loss of MHC-II fundamentally alters that program. It will be important to define the signaling pathway(s) controlled by MHC-II function.

We found that fewer pTreg, and therefore less suppressive activity, were present in tumors in mixed *H2-Aa*^*cit/cit*^ + WT bone marrow chimeras than in chimeras with WT + WT BM, although Treg frequencies in the spleen were similar. Conversely, DC infiltration into tumors was greater in *H2-Aa*^*cit/cit*^ mice than in WT mice. These effects may result from the loss in *H2-Aa*^*cit/cit*^ mice of brief, unstable MHC-II–mediated interactions between cDC and Treg, interactions which would normally promote Treg function and expansion over those of T helper cells in the tumor microenvironment (Marangoni et al., 2021). At the same time, the reduction in tumor Treg prevents the downregulation of costimulatory B7-family proteins (CD80 and CD86) on cDC in a CTLA-4-dependent manner (Qureshi et al., 2011; Wing et al., 2008). Thus, the reduction of Treg specifically in the tumor microenvironment facilitates cDC cross-priming activity toward CD8 T cells in *H2-Aa*^*cit/cit*^ mice.

Our results showed that MHC-II monoclonal antibody treatment successfully blocked melanoma growth in mice, leading us to hypothesize that antibodies blocking HLA class II might achieve similar tumor suppression in humans by empowering cDC2-mediated priming of CD8 T cells that recognize tumor-specific antigens. We wish to point out that transient blockade of HLA class II might not only achieve a therapeutic effect but could, in individual patients, be used to reveal precisely which tumor-directed TCRs undergo expansion and therefore, which TCRs are presumptively therapeutic. The therapeutic effect of HLA class II blockade could likely be augmented by checkpoint inhibitor treatment, just as it clearly was in our mouse model. Unlike C57BL/6J mice, which express only a single type of MHC-II (IA^b^, with α and β chains encoded by *H2-Aa* and *H2-Ab1*, respectively), human MHC class II molecules (HLA class II) are encoded by three pairs of polymorphic genes: HLA-DR, HLA-DQ (orthologous to IA^b^) and HLA-DP α and β genes (Neefjes et al., 2011; Roche and Furuta, 2015). Considering the highly polymorphic nature of HLA class II, it is not clear whether antibody-mediated blocking of HLA-DQ by itself or in combination with HLA-DR and HLA-DP would universally achieve tumor resistance as achieved in C57BL/6J mice with HB12-18 treatment. It is, on the one hand, possible that even a partial blockade of HLA class II will achieve tumor suppression based on our finding that a mixture of fully functional WT DC and *H2-Aa*^*cit/cit*^ DC was able to inhibit melanoma growth. Alternatively, targeting CIITA might be a better choice. CIITA regulates both the constitutive and the inducible expression of HLA class II genes (Forlani et al., 2023). A CIITA inhibitor might be more useful in humans than antibodies against HLA class II proteins because of the abundance of these proteins, their relatively rapid turnover, and the difficulties presented by polymorphism.

In conclusion, our study has identified H2-Aa as a protein that normally limits cDC2 development and cross-priming ability, effectively encouraging the progression of melanoma growth by preventing a robust CD8 T cell response. Blockade of H2-Aa enhances CD8 T cell activation. Both *H2-Aa* mutations (whether constitutive or induced) and antibody-mediated H2-Aa blockade synergize with immune checkpoint inhibition. *Ciita* mutation (whether constitutive or induced) does so as well. These findings suggest novel immunotherapeutic opportunities.

## Materials and methods

### Mice

8–12-wk-old male and female mice (*Mus musculus*) were used in experiments. Male C57BL/6J mice purchased from The Jackson Laboratory were mutagenized with ENU as described (Wang et al., 2015). Mutagenized G0 males were bred to C57BL/6J females, and the resulting G1 males were crossed to C57BL/6J females to produce G2 mice. G2 females were backcrossed to their G1 sires to yield G3 mice, which were screened for phenotypes. Whole-exome sequencing and mapping were performed as described (Wang et al., 2015). The *H2-Aa*^*cit*^ mutant strain was generated by mutagenesis with ENU. *MHC-II* KO (003584), C57BL/6.SJL (CD45.1, 002014), BALB/cJ (000651), *Rag2* KO (008449), Cd11c-Cre (008068), Xcr1-Cre (035435), Cd19-Cre (006785), LysM-Cre (004781), UBC-Cre-ER^T2^ (007001), and Foxp3-DTR mice (016958) were purchased from The Jackson Laboratory. All experimental procedures using mice were approved by the Institutional Animal Care and Use Committee of the University of Texas Southwestern Medical Center, and were conducted in accordance with institutionally approved protocols and guidelines for animal care and use.

### Cell culture

B16F10 (CRL-6475) and YUMM1.G1 (CRL-3363) cells were purchased from ATCC. MC38 (CVCL-B288) cells were purchased from Kerafast. These tumor cell lines were derived from the C57BL/6 mouse strain. B16F10-OVA cells (B16F10 melanoma cells stably expressing chicken OVA) were a gift from Dr. Yang-Xin Fu (UT Southwestern, Dallas, TX, USA). Cells were grown in Dulbecco’s modified Eagle’s medium (DMEM; Gibco) supplemented with 10% fetal bovine serum (FBS; ATCC) and 1% penicillin–streptomycin (Gibco) at 37°C in 5% CO_2_.

Primary T cells and DC were grown in RPMI 1640 (Gibco) Full Medium containing 10% FBS (ATCC), 1% penicillin–streptomycin, 25 mM HEPES (Gibco), 1 mM sodium pyruvate (Gibco), 50 μM 2-mercaptoethanol (Gibco), and 1% MEM non-essential amino acids (100×; Gibco) at 37°C in 5% CO_2_.

### Generation of *H2-Aa*^*flox*^ mice and *H2-Aa* KO B16F10 cells

CRISPR-Cas9–mediated gene targeting was used to generate mice with the *H2-Aa*^*flox*^ allele, in which exon 1 was flanked by loxP sites. Female C57BL/6J mice were superovulated by injection of 6.5 U pregnant mare serum gonadotropin (Millipore), followed by injection of 6.5 U human chorionic gonadotropin (Sigma-Aldrich) 48 h later. The superovulated mice were subsequently mated overnight with C57BL/6J male mice. The following day, fertilized eggs were collected from the oviducts and injected into the cytoplasm or pronucleus with in vitro–transcribed Cas9 mRNA (50 ng/μl), *H2-Aa* upstream small base-pairing guide RNA (50 ng/μl; 5′-TCT​GTG​GGG​ACA​CCT​TGG​GT-3′), upstream ssODN template (50 ng/μl; 5′-TGC​TTG​CAT​GCA​TCA​TGA​GTT​AGC​TGG​TTT​TTC​TTT​ATT​TCT​TCC​CAT​AGT​GTG​TGC​AGA​GTA​CTC​CAA​ATT​GTG​AGA​TGA​TCC​ACC​TAC​CAT​AAC​TTC​GTA​TAG​CAT​ACA​TTA​TAC​GAA​GTT​ATG​GAT​CCC​AAG​GTG​TCC​CCA​CAG​ACT​GTG​GTC​AGT​CAA​TGG​CTT-3′), downstream small base-pairing guide RNA (50 ng/μl; 5′-CGG​TGC​TAA​GGT​GCC​CAA​GC-3′), and downstream ssODN template (50 ng/μl; 5′-CAT​TTA​AAG​CAT​TTA​CTA​ATG​AAT​GGA​TAC​ATT​CTT​GAG​ACA​TGA​TCT​CAC​TAG​GGG​ATC​AGG​CTA​TCC​TTG​AAC​TCA​CAA​TCC​TCC​TGC​TCT​CGA​GAT​AAC​TTC​GTA​TAG​CAT​ACA​TTA​TAC​GAA​GTT​ATT​GGG​CAC​CTT​AGC​ACC​GTA​GTT​ACT​GCC​AAG​CAC​TGC​C-3′). The injected embryos were cultured in M16 medium (Sigma-Aldrich) at 37°C in 95% air plus 5% CO_2_. To produce mutant mice, two-cell stage embryos were transferred into the ampulla of the oviduct (10–20 embryos per oviduct) of pseudopregnant Hsd:ICR (CD-1) female mice (Harlan Laboratories). Chimeric mutant mice were crossed with C57BL/6J mice and their offspring were intercrossed to produce mice homozygous for the floxed allele (*H2-Aa*^*flox/flox*^). The *H2-Aa*^*flox/flox*^ mice were genotyped by capillary sequencing with the following primers: (5′ loxP site) 5′-AGA​GTG​CCT​GGG​AAG​AAG​TGG-3′ and 5′-TCT​TGT​GTG​TGT​TCA​GTG​TCC​C-3′ as the PCR primers, and 5′-GGG​AAG​AAG​TGG​GTT​CTA​AG-3′ as the sequencing primer; (3′ loxP site) 5′-GCC​TGG​GTT​GTT​ACA​GAA​CTT​CA-3′ and 5′-ATA​CAG​CAA​ATG​CTT​CCC​TTG​TG-3′ as the PCR primers and 5′-TCA​TGG​GGT​ATA​AAT​GGC​CT-3′ as the sequencing primer. For the generation of *H2-Aa* KO B16F10 cells, cells were infected with lentivirus (lentiCRISPR v2) encoding Cas9 and the sgRNA 5′-ACC​ACC​ATG​CTC​AGC​CTC​TG-3′ targeting *H2-Aa* exon1. After puromycin selection, single cells were selected for subculture and confirmed by capillary sequencing.

### Tumor inoculation and tumor growth assay

B16F10 (2 × 10^5^ in 100 μl PBS), B16F10-OVA (5 × 10^5^ in 100 μl PBS), YUMM1.G1 (1 × 10^6^ in 100 μl PBS), or MC38 (1 × 10^6^ in 100 μl PBS) were subcutaneously (s.c.) injected into the flank of mice. For monoclonal antibody HB12-18 treatment, mice were injected intraperitoneally (i.p.) with the indicated amounts of antibodies after tumor inoculation. For anti-PD1 treatment, mice were injected i.p. with 10 μg/g body weight anti-PD1 (RMP1-14; BioXCell) in 200 μl PBS on days 5, 8, and 11, unless otherwise stated after tumor inoculation. For depletion of CD4 T cells, CD8 T cells, or NK cells, 300 μg anti-mCD4 (GK1.5; BioXcell), anti-mCD8 (YTS 169.4; BioXcell), or anti-mNK1.1 (PK136; BioXcell) in 200 μl PBS were injected i.p. into mice on days 0, 3, 6, 9, 12, and 15 after tumor inoculation. For intratumoral injection, FACS-sorted cDC2 (2 × 10^5^ in 50 μl PBS) were injected on day 3 after tumor inoculation. Tumor volumes were calculated using length and width measurements and the following formula: volume = 0.5 × length × width^2^. Tumor volumes were plotted over time. Mice were euthanized when the tumor length reached 20 mm. The tumor size limit was approved by the Institutional Animal Care and Use Committee of the University of Texas Southwestern Medical Center. For intravenous (i.v.) inoculation, mice were injected with 1 × 10^5^ B16F10 cells via tail vein on day 0; this experimental metastasis model results in metastatic melanoma foci in the lungs. They were monitored for survival on a daily basis after inoculation.

### Bone marrow chimeras

Recipient WT (CD45.1) or *H2-Aa*^*cit/cit*^ *(cit,* CD45.2*)* mice were lethally irradiated with 12 Gy using 320-kV X-rays and injected i.v. 2–3 h later with 5 × 10^6^ BMC collected from the tibias and femurs of the respective donors. 3 mo after engrafting, the chimeras were assessed using flow cytometry analysis of specific cell populations as indicated and using tumor measurements after s.c. injection with B16F10 tumor cells.

### Flow cytometry

Cells from bone marrow, lymph nodes, spleens, or peripheral blood were isolated, and RBC lysis buffer (eBioscience) was added to remove the RBCs. Samples were washed with FACS staining buffer (PBS with 1% [wt/vol] bovine serum albumin) one time at 500 × *g* for 5 min. The RBC-depleted samples were stained for 1 h at 4°C in 100 μl of a cocktail containing fluorescence-conjugated antibodies to 15 cell surface markers encompassing the major immune lineages (diluted 1:200): B220 (clone RA3-6B2; BD), CD19 (clone 1D3; BD), IgM (clone R6-60.2; BD), IgD (clone 11-26c.2a; BioLegend), CD3ε (clone 145-2C11; BD), CD4 (clone RM4-5; BD), CD8α (clone 53-6.7; BioLegend), CD11b (clone M1/70; BioLegend), CD11c (clone HL3; BD), F4/80 (clone BM8.1; Tonbo), CD44 (clone 1M7; BD), CD62L (clone MEL-14; Tonbo), CD5 (clone 53-7.3; BD), MHC-II (clone M5/114.15.2; BioLegend), NK1.1 (clone OK136; BioLegend), and 1:200 Fc block (clone 2.4G2; Tonbo). After staining, cells were washed twice with FACS staining buffer and analyzed by flow cytometry. To stain DC, the following antibodies were used: lineage markers (CD3, CD19, NK1.1, and Ter-119 [363-5921-8; eBioscience]), CD11b, CD11c, CD8α, Xcr1 (clone ZET; BioLegend), Sirpα (clone P84; BioLegend), Ly6C (clone HK1.4; BioLegend), CD80 (clone 16-10A1; BioLegend), MHC-I (clone 28-8-6; BioLegend), MHC-II, ESAM (clone 1G8; BioLegend), and Clec12A (clone 5D3; BioLegend). For cell counting, an equal amount of counting beads (Invitrogen) was added to each sample during staining. To stain the progenitors of DC, bone marrow was isolated and stained with antibodies against lineage markers (CD3, CD19, NK1.1, and Ter-119), CD11c, Flt3 (clone A2F10; eBioscience), Sirpα, Ly6C, and Siglec-H (clone 551; BioLegend) at a 1:200 dilution for 1 h at 4°C. For intracellular staining, cells were first stained with Zombie UV fixable viability dye and antibodies for surface antigens before being fixed with PBS containing 2% paraformaldehyde and then permeabilized with Perm Buffer (Foxp3/Transcription Factor Staining Buffer Set) containing the intracellular antibodies. Data were acquired on an LSRFortessa cell analyzer (BD Biosciences) and analyzed with FlowJo software (BD Biosciences). For flow sorting of cDC subsets, DC were first enriched from tissue cell suspensions using EasySep Mouse CD11c Positive Selection Kit II (Stemcell) according to the manufacturer’s instructions.

### TIL separation and staining

Tumors were harvested, minced, digested with 1 mg/ml collagenase D (Sigma-Aldrich) and 0.2 mg/ml Dnase I (Sigma-Aldrich) at 37°C for 30 min, and filtered through a 40-μm strainer to obtain single-cell suspensions. After pelleting, CD45^+^ cells were stained for 45 min at 4°C with DC staining antibody panel as listed above, or Treg staining antibody panel containing antibodies against mouse CD45.1 (clone A20; BioLegend), CD45.2 (clone 104; BioLegend), CD3, CD4, CD8, CD44, CD62L, CD25 (clone PC61; BioLegend), Nrp1 (clone 3E12; BioLegend), and NK1.1. Then the cells were washed twice with FACS staining buffer. Stained cells were analyzed with an LSRFortessa cell analyzer (BD Biosciences), and the flow cytometry data were analyzed using FlowJo software (BD Biosciences).

### *H2-Aa* RT-PCR

Total RNA was isolated from splenocytes using the Rneasy RNA extraction kit (Qiagen), and cDNA synthesis was performed using 1 μg of total RNA (iScript; Bio-Rad). PCR was performed with the following gene-specific primers: *H2-Aa*, 5′-ATG​CCG​CGC​AGC​AGA​GCT​CTG​ATT​C-3′ (fwd) and 5′-TCA​TAA​AGG​CCC​TGG​GTG​TCT​GGA​G-3′ (rev).

### Measurement of cytokines

Cytokine concentrations in the culture medium (IFNγ secreted by cultured OT-I CD8 T cells) or peripheral blood plasma were measured with V-PLEX Proinflammatory Panel 1 Mouse Kit (Meso Scale Diagnostics) according to the manufacturer’s instructions.

### BMDC culture and antigen uptake assay

Single-cell suspensions of BMC were cultured in RPMI 1640 medium containing 10% heat-inactivated FBS. For GM-CSF–induced DC, on days 3 and 5, the medium was replaced with fresh medium containing GM-CSF (20 ng/ml; Peprotech). On day 6, GM-CSF–induced BMDCs were purified using the EasySep Mouse CD11c Positive Selection Kit II (Stemcell). For Flt3L-induced DC, single-cell suspensions of BMCs were cultured in a medium with 100 ng/ml Flt3L (Peprotech) for 9 days to obtain Flt3L-BMDC.

BMDCs (2 × 10^5^ cells/ml) were incubated with FITC-labeled OVA (100 μg/ml; Vivitide) for 1 h or with 50 Gy irradiated and CTV (1 μM; Invitrogen)-labeled B16F10 (2 × 10^5^ cells/ml) for 4 h at 37°C to assess antigen uptake. The uptake of FITC-OVA and CTV-B16F10 was assessed by measurement of the mean fluorescence intensity (MFI) of FITC and CTV, respectively, using flow cytometry.

### In vitro cross-priming assay

5 × 10^5^ B16F10-OVA cells per mouse were s.c injected into the flank of WT and *H2-Aa*^*cit/cit*^ mice. 6 days after tumor cell inoculation, draining lymph nodes were collected and applied for FACS sorting. 5 × 10^4^ CTV-labeled naïve OT-I CD8 T cells enriched from spleens using EasySep Mouse CD8^+^ T Cell Isolation Kit (Stemcell) were co-cultured with 1 × 10^4^ purified cDC1 (Lin− CD45^+^ Ly6C− CD11c+ Xcr1+ CD11b−), cDC2 (Lin− CD45^+^ Ly6C− CD11c+ Xcr1− CD11b+), or macrophages (Lin− CD45^+^ CD11b+ F4/80+) in 200 μl RPMI 1640 Full Medium in U-shaped-bottom microplates (Falcon) at 37°C in 5% CO_2_. 3 days later, OT-I CD8 T cell proliferation indicated by CTV dilution was analyzed by flow cytometry.

### In vivo cross-priming assay

On day 0, CTV (1 μM; Invitrogen)-labeled naïve OT-I CD8 T cells (CD45.2) (together with CTF [0.5 μM; Invitrogen]-labeled naïve OT-II CD4 T cells [CD45.2] when testing HB12-18 [Fig. S5 D]) were injected (1 × 10^6^ cells/mouse) i.v. into indicated recipient mice. The next day (day 1), recipient mice were immunized i.p. with soluble OVA (VacciGrade, 1 mg/mouse; Invivogen), and splenocytes were isolated for analysis on day 4. APC-labeled H2-K^b^ OVA tetramer (SIINFEKL, MHC Tetramer Production Core, Baylor College of Medicine, Houston, TX, USA) was used to gate all the OT-I CD8 T cells (APC positive) in the CD45.2 WT and *H2-Aa*^*cit/cit*^ recipient mice. Counting beads (Invitrogen) were added during flow cytometry analysis.

### ScRNA-seq

Splenic DC were enriched from splenocyte suspensions using EasySep Mouse CD11c Positive Selection Kit II (Stemcell) according to the manufacturer’s instructions. Then, about 1.5 × 10^4^ live CD45.2+ CD11c+ cells were FACS-sorted from WT or *H2-Aa*^*cit/cit*^ DC enriched samples. scRNA-seq libraries were generated using the Chromium Next GEM Single Cell 3′ kit (10X Genomics) according to the manufacturer’s protocol. Purified libraries were sequenced on one S4 lane of a NovaSeq sequencing instrument with the run configuration 150 × 10 × 10 × 150.

### ScRNA-seq analysis

Cell Ranger (v5.0.1; 10X Genomics) mkfastq module was used to convert BCL files to FASTQ format. Reads from FASTQ files for each library were aligned to the mouse reference genome (GRCm38) and transcript counts of each cell were quantified using UMI and a valid cell barcode. The gene expression matrix generated from the Cell Ranger count module was then used as input to the Seurat R package (v4.0.1) for the downstream analysis (Hao et al., 2021). Cells with <200 genes per cell and very high mitochondrial gene content were filtered out. Sctransform was used for the normalization and variance stabilization of UMI count data (Hafemeister and Satija, 2019). Briefly, the model calculates the Pearson residuals representing variance-stabilization transformation reducing dependence between the gene’s average expression and cell-to-cell variations, thus removing technical variations and preserving biological heterogeneity. FindIntegrationAnchors and IntegrateData modules in Seurat were used to find anchors and integrate Seurat objects corresponding to *H2-Aa*^*cit/cit*^ and WT DC samples. Seurat integrated analysis was performed across *H2-Aa*^*cit/cit*^ and WT samples. Data was then scaled and dimensional reduction was performed with principal component analysis. For the sample, a shared nearest neighbor (SNN) graph was constructed with the FindNeighbors module in Seurat by determining the k-nearest neighbors of each cell. The clusters were then identified by optimizing SNN modularity using the FindClusters module. This allowed for sensitive detection of rare cell types. We obtained 10 clusters with a resolution of 0.2. UMAP plots were generated using the DimPlot module in Seurat. Each cluster was compared to all other clusters using the Wilcoxon rank sum test to test for significant differentially expressed genes. The genes identified as relatively overexpressed in a cluster as compared with all other cells were termed as “markers.” Clusters were named based on gene markers specific to various cell types. Differential gene expression testing was performed between clusters in *H2-Aa*^*cit/cit*^ and WT DC samples using the FindMarkers module in Seurat.

### Tamoxifen treatment for Cre induction

Tamoxifen (Sigma-Aldrich) was solubilized at 20 mg/ml in corn oil by shaking overnight at 37°C. It was delivered into mice by i.p. injection (100 mg/kg body weight, once per day for 3 days).

### Generation of monoclonal antibodies against MHC-II

BALB/cJ mice were immunized with C57BL/6J MHC-II by grafting C57BL/6J skins onto them. 4 wk after skin grafting, BALB/cJ splenocytes were collected and fused with Sp2/0-Ag14 cells (CRL-1581; ATCC) using ClonaCell-HY Hybridoma Kit (Stemcell) according to the manufacturer’s protocol. The fused cells were plated in a semi-solid selection medium and clonal outgrowths were harvested into single wells of 96-well plates after 10–14 days. Single clones were allowed to grow for 4–6 days in suspension culture after which the supernatant was tested for reactivity against MHC-II. Specifically, 1 × 10^5^ splenocytes from WT or *H2-Aa*^*cit/cit*^ mice were incubated with supernatants from different hybridomas for 30 min at 4°C. After washing, the cells were stained with anti-CD19, anti-IgG1 (Clone A85-1; BD), and anti-IgG2 (Clone R2-40; BD) for 30 min at 4°C and analyzed by flow cytometry. Supernatants containing IgG1 or IgG2 antibodies reactive with WT B cells but not with *H2-Aa*^*cit/cit*^ B cells contained putative monoclonal antibodies against MHC-II. 5 × 10^4^ BALB/3T3 cells (CCL-163; ATCC) and 5 × 10^4^ BALB/3T3 cells stably expressing C57BL/6 MHC-II (*H2-Aa* and *H2-Ab1*) were used to further validate the specific recognition of MHC-II by putative monoclonal antibodies following a similar process as used to stain the splenocytes. The monoclonality of the hybridomas was verified by sequencing. Small-scale quantities of antibodies were purified using a Hi-Trap Protein G column (Cytiva). Large amounts of HB12-18 were produced by Thermo Fisher Scientific based on the DNA sequence we provided.

### Immunoblotting

Cells were lysed in RIPA buffer (50 mM Tris-Cl, pH 7.5, 150 mM NaCl, 1 mM EDTA, 1 mM EGTA, 1% vol/vol NP-40, 0.1% wt/vol SDS, 1 mM dithiothreitol, plus Halt Protease, and Phosphatase inhibitor Cocktail [Thermo Fisher Scientific]) immediately before use, and protein contents were determined by BCA assay (Thermo Fisher Scientific). Equal amounts (∼20 μg) of protein extracts were separated by electrophoresis on 4–12% NuPAGE Bis-Tris Mini Gels (Invitrogen) and then transferred to nitrocellulose membranes (Bio-Rad). The membrane was blocked for 1 h in Tris-buffered saline containing 0.1% vol/vol Tween 20 and 5% wt/vol non-fat milk and then probed with various primary antibodies overnight, followed by secondary antibodies conjugated to horseradish peroxidase. The immunoreactivity was detected with SuperSignal West Dura Chemiluminescent Substrate (Thermo Fisher Scientific). Antibodies for Nlrc5 (B-10; Santa Cruz), β-actin (A2228), and α-tubulin (T6199) (Sigma-Aldrich) were used.

### Statistical analysis

The statistical significance of differences between experimental groups was determined using GraphPad Prism 10 and the indicated statistical tests. For comparisons of differences between two unpaired experimental groups, an unpaired Student’s *t* test was used. Two-way ANOVA with post-hoc Tukey test was applied to experiments with three or more groups; for tumor growth curves, P values shown correspond to the latest timepoint. For comparison of survival distributions, the log-rank test was used. The P values of non-association between genotype and phenotype detected in the screening of ENU-mutagenized mice were calculated using a likelihood ratio test from a generalized linear model or generalized linear mixed effect model and Bonferroni correction applied (Wang et al., 2015). P ≤ 0.05 was considered statistically significant.

### Online supplemental material

Fig. S1 shows the melanoma resistance phenotype of *MHC-II* KO mice and whole blood cell counts and plasma cytokine concentrations in WT and *H2-Aa*^*cit/cit*^ mice. Fig. S2 shows the cross-priming activity of *H2-Aa*^*cit/cit*^ macrophages and bone marrow–derived cDC2, numbers, and frequencies of splenic cDC1 in *H2-Aa*^*cit/cit*^ mice and *H2-Aa*^*flox/flox*^;Xcr1-cre mice, and DC development in the bone marrow and cDC1/2 in MLN in *H2-Aa*^*cit/cit*^ mice. Fig. S3 shows scRNA-seq analysis of splenic DCs from WT and *H2-Aa*^*cit/cit*^ mice. Fig. S4 shows cDC1/2 frequencies and MHC-I expression and lymphocyte frequencies in different tissues in WT+*H2-Aa*^*cit/cit*^ mixed bone marrow chimeric mice, and the effect of intratumoral injection of *H2-Aa*^*cit/cit*^ cDC2 on B16F10 tumor growth. Fig. S5 shows the specificity and effect on cross-priming of the MHC-II monoclonal antibody HB12-18. Data S1 shows raw scRNA-seq clustering of splenic cDC from WT and *H2-Aa*^*cit/cit*^ mice. Data S2 shows fold changes of genes in splenic cDC2 subtypes from *H2-Aa*^*cit/cit*^ versus WT mice by scRNA-seq.

## Supplementary Material

Data S1 shows raw scRNA-seq clustering of splenic cDC from WT and *H2-Aa*^*cit/cit*^ mice.

Data S2 shows fold changes of genes in splenic cDC2 subtypes from *H2-Aa*^*cit/cit*^ versus WT mice by scRNA-seq.

SourceData F4 is the source file for Fig. 4.

## Data Availability

The single-cell RNA sequencing data underlying Fig. 4, G–J, and Fig. S3 are openly available from the NCBI GEO database with accession no. GSE266960.
