# Supplementary figures and images for: Suppression of melanoma by mice lacking MHC-II: Mechanisms and implications for cancer immunotherapy
Source: J Exp Med. 2024 Oct 29;221(12):e20240797. doi: 10.1084/jem.20240797 (PMC11528124; doi:10.1084/jem.20240797)

Fig. 4F

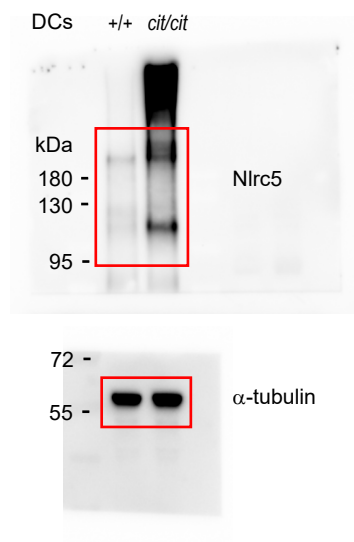

Supplement: SourceData F4 — is the source file for Fig. 4. [file JEM_20240797_SourceDataF4.pdf]
